# Kidney-targeted rhein-loaded liponanoparticles for diabetic nephropathy therapy via size control and enhancement of renal cellular uptake

**DOI:** 10.7150/thno.37538

**Published:** 2019-08-14

**Authors:** Guowei Wang, Qunying Li, Danfei Chen, Bihan Wu, Yulian Wu, Weijun Tong, Pintong Huang

**Affiliations:** 1 Department of Ultrasound in Medicine, The Second Affiliated Hospital of Zhejiang University School of Medicine, Zhejiang University, Hangzhou 310009, China.; 2 Department of Pediatrics, The First Affiliated Hospital of Zhejiang Chinese Medical University, Zhejiang Chinese Medical University, Hangzhou, 310006 China.; 3 MOE Key Laboratory of Macromolecular Synthesis and Functionalization, Department of Polymer Science and Engineering, Zhejiang University, Hangzhou 310027, China.

**Keywords:** diabetic nephropathy, rhein, liponanoparticles, size-control, kidney-targeted drug delivery

## Abstract

The optimization of nanoparticle size for passing through glomerular filtration membrane, inefficient renal cellular uptake and rapid urinary excretion of nanoparticles are the major obstacles for renal disease treatment via a nanoparticle delivery system. Herein, we propose a concept of a two-step nanoparticular cascade of size control and enhancement of renal cellular uptake to overcome the renal delivery obstacles.

**Methods**: We prepared kidney-targeted rhein (RH)-loaded liponanoparticles (KLPPR) with a yolk-shell structure composed by polycaprolactone-polyethyleneimine (PCL-PEI)-based cores and kidney targeting peptide (KTP)-modified lipid layers. The KLPPR size within the range of 30 ~ 80 nm allowed KLPPR distribute into kidney by passing through the glomerular filtration membrane and the KTP (sequence: CSAVPLC) decoration promoted the renal cellular uptake and endocytosis via a non-lysosomal pathway.

**Results**: The KLPPR had an average size of 59.5±6.2 nm and exhibited high RH loading, sustained release, good stability and biocompatibility, rapid cellular uptake in HK-2 cells. In addition, intravenous administration of KLPPR resulted in excellent kidney-targeted distribution and low urinary excretion in mice with streptozocin-induced diabetic nephropathy (DN), lowered the parameters of urea nitrogen, serum creatinine and kidney index, as well as facilitated the recovery of renal physiological function in improving the levels of urinary creatinine and the creatinine clearance rate by suppressing secretion and accumulation of fibronectin and TGF-β1.

**Conclusion**: Definitely, KLPPR were able to target the diseased kidney and improve the therapeutic effect of RH on DN by exploiting the two-step nanoparticular cascade of size control and enhancement of cellular uptake. This study offers a promising strategy for renal diseases treatment using liponanoparticle delivery system.

## Introduction

Diabetic nephropathy (DN), one of the most common and life-threatening complications of diabetes, is the foremost cause of end-stage renal failure and death of both type I and II diabetic patients 1, 2. As genetic problems, early onset of puberty, heightened diabetes risks and unhealth diet habits have increased, DN has been emerging as a worldwide epidemic disease 1, 3. According to statistics, nearly 425 million people had diabetes in 2017 4, and this number is expected to increase to 592 million by 2035 in the worldwide 3. Unfortunately, clinical therapies or medications that can efficiently alleviate or reverse the deterioration of DN are currently not available.

Rhein (RH) is an active ingredient separated from *Rheum palmatum L*., *R. tanguticum Maxim. ex Balf.*, and other herbal medicines and has drawn wide attention in Asian countries for its anti-diabetic activities 5, 6. In addition, the use of RH capsules has been approved for Phase II clinical trials in China (clinical trial approval number: 2008L03643). Pharmacological studies have confirmed that RH markedly suppressed the proliferation of glomerular mesangial cells, hypertrophy of glomerulus, and the production and accumulation of extracellular matrix in DN patients and mice 5-7. Moreover, RH can protect the intrinsic function of kidney by inhibiting mRNA transcription and the expression of thrombospondin-1 (TSP-1) and transforming growth factor-beta1 (TGF-β1) in renal tubular epithelial cells and decreasing kidney inflammation 8, 9. Thus, RH plays a multi-target and multi-level role in DN therapy. However, clinical applications of RH are hampered by its poor solubility, short half-life, low renal accumulation, poor bioavailability and hepatotoxic side-effects.

Nanoparticles, which are highly suitable for loading and delivering poorly soluble drugs (e.g. paclitaxel, doxorubicin, 10-hydroxycamptothecin), are capable of improving bioavailability, maintaining long-term blood circulation and achieving nidus-targeted distribution, and their use has resulted in great success especially in anti-tumor therapy due to enhanced permeability and retention effect 10, 11. A design criterion for constructing nanoparticle-based therapeutics for kidney diseases is controlling the size of nanoparticles into a specific region due to the innate ability of kidneys to select nanoparticles of a certain sizes. Specifically, nanoparticles pass through the glomerular filtration membrane if they are smaller than 10 nm, whereas those with sizes larger than 100 nm seldom distribute into kidney because they are mostly trapped by liver and spleen 12-14. In sharp contrast, nanoparticles with sizes of 30 ~ 80 nm can be sequestered by the mesangium of kidney in DN, thereby achieving reduced liver retention and hepatotoxicity 14-16. In our previous work, nanoparticles with a size of 75.4 ± 2.9 nm exhibited specific kidney distribution in DN model mice 16. To date, several reports have demonstrated RH-loaded nanoparticle delivery system for improving solubility and blood circulation time 17, 18, but an efficient nanoparticle targeted delivery for DN therapy remains under investigation. Although the feasibility of using nanoparticles in kidney-targeted distribution via optimal size-control and mesangial filtration has been demonstrated, the nanoparticles will be excreted in urine quickly if they fail to be internalized rapidly by renal cells. Moreover, most of renal cells in patients with DN, especially the renal tubular cells, exhibit cellular uptake dysfunctions of glucose, protein and mineral salt, as well as nanoparticles 12, 19, which has hampered improvements in the therapeutic effect related to the nanoparticles. Thus, efforts are still needed to overcome the obstacles of inefficient cellular uptake by renal cells and rapid urinary excretion of nanoparticles in DN therapy.

**Scheme 1:**
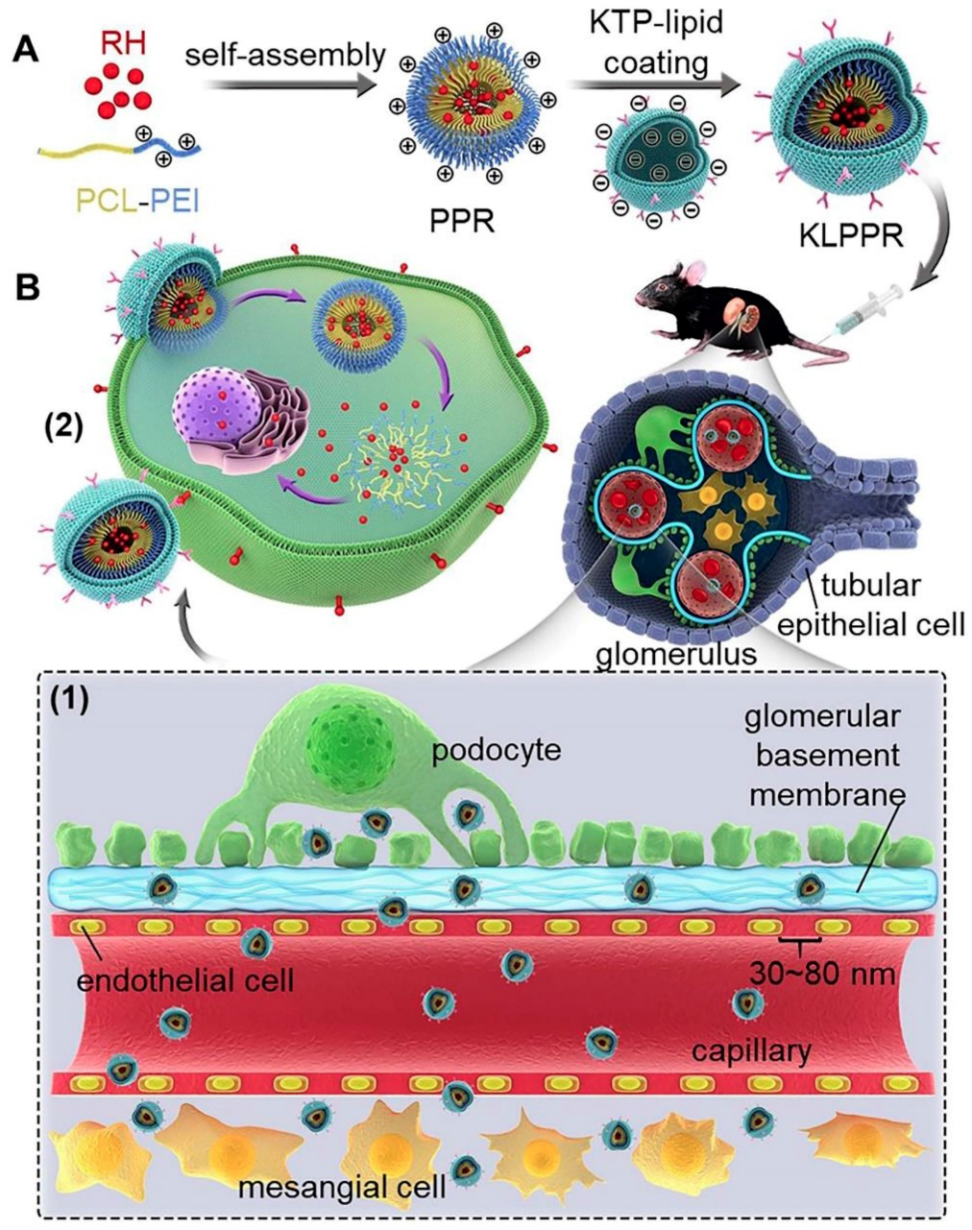
** Schematic representation of KLPPR liponanoparticles for kidney-targeted drug delivery.** (A) KLPPR liponanoparticles consisted of a RH-loaded PCL-PEI nanoparticular core and a KTP-modified lipid layer were created, based on the principle of electrostatic interaction between the electropositive core and electronegative lipid layer. (B) The concept of the two-step nanoparticular cascade including size control and enhancement of renal cellular uptake. Maintaining the size in the range of 30 ~ 80 nm allowed KLPPR enter kidney by passing through the glomerular filtration membrane (1); KTP decoration promoted the renal cellular uptake and internalization via membrane fusion and improved the kidney retention of KLPPR (2).

Liponanoparticles are a self-assembled yolk-shell nanocarrier system resulting from electrostatic or hydrophobic interactions between the outer lipid shell and the inner nanoparticles core in aqueous solutions. They possess the advantages of high drug loading efficiency, good stability and facile modification of lipid layers 20, 21. Kidney targeting peptides (KTP) with the unique sequence of CSAVPLC are elastin-like peptides and have been proved to effectively recognize and promote endocytosis of renal cells 19, 22, 23. Previous studies have shown that KTP-modified prodrugs can result in noticeable improvements in the drug delivery to kidneys and quick cellular internalization of the renal tubular cells, endothelial cells, mesangial cells, and podocytes 19, 23. In this regard, we propose a concept of two-step nanoparticular cascade including size control and enhancement of renal cellular uptake to provide safe and efficient treatment for DN. The size maintenance of liponanoparticles in the range of 30 ~ 80 nm allows them to pass through the glomerular filtration membrane and the KTP decoration promotes the renal cellular uptake and internalization by elastin-like peptides-based ligand/receptor recognition (Scheme 1).

Herein, polycaprolactone-polyethyleneimine (PCL-PEI) and RH-loaded PCL-PEI nanoparticles (PPR) were synthesized. After coating with a KTP-modified 1,2-distearyl-sn-glycerol-3-phosphoethanolamine-polyethylene glycol (DSPE-PEG-KTP) lipid layer, the RH-loaded KTP-modified liposomal PCL-PEI nanoparticles (KLPPR) were developed via electrostatic interaction between the electropositive PPR core and electronegative lipid layer (Scheme 1). The preparation methods for KLPPR were optimized and screened to obtain optimal encapsulation efficiency, drug loading efficiency, and particle size. Pharmaceutical properties, biocompatibility, cytotoxicity, cellular uptake, and subcellular distribution of KLPPR *in vitro* were characterized in detail. *In vivo* stability, blood clearance, kidney-targeted biodistribution, and pharmacodynamics of KLPPR were further investigated in streptozotocin (STZ)-induced DN mice.

## Materials and Methods

### Materials

Kidney targeting peptides (sequence: lysine-cysteine-serine-alanine-valine-proline- leucine-cysteine, H_2_N-KCSAVPLC, purity ≥ 95%) were synthesized and purified by China Peptides Co., Ltd (Shanghai, China). The RH active pharmaceutical ingredient (purity ≥ 98%) was purchased from Zelang Medical Technology Co. Ltd. (Nanjing, China). Lantus (insulin glargine, a long-acting basal insulin analogue) was purchased from Sanofi-Aventis (France). 1,2-Dioleoyl-sn-glycero-3-phosphoethanolamine (DOPE), cholesteryl hemisuccinate (CHEMS), and 1,2-distearyl-sn-glycerol-3-phosphoethanolamine-polyethylene glycol 2000 (DSPE-PEG) were purchased from Avanti Lipids Inc. (USA). 1,2-Distearyl-sn-glycerol-3-phosphoglycolamine-polyethylene glycol 2000-n-hydroxysuccinimide (DSPE-PEG-NHS) was purchased from Laysan Bio. Inc (USA). Cy5.5-labeled DSPE-PEG (DSPE-PEG^Cy5.5^) was purchased from Xi'an Ruixi Biological Technology Co., Ltd (Xi'an, China). 2-methyl-2-oxazoline, methyl p-toluenesulfonate, dicyclohexylcarbodiimide (DCC), and N-hydroxysuccinimide (NHS) were purchased from Energy Chemical (Shanghai, China). Carboxyl terminated PCL (MW: 3.7 kDa) was purchased from Jinan Daigang Biomaterial Co., Ltd (Jinan, China). 3-(4,5-Dimethylthiazol-2-yl)-2,5-diphenyl tetrazolium bromide (MTT) and streptozotocin (STZ) were purchased from Aldrich Chemical Co., Ltd (Shanghai, China). Cyanine5 n-hydroxysuccinimide active ester (Cy5-NHS) was purchased from Mercury Bio-pharmaceutical Technology Co. Ltd (Hangzhou, China). Anti-TGF-β1 antibody and anti-Smad2/3 antibody for Western blotting were purchased from Abcam (UK). Anti-fibronectin antibody for immunohistochemistry (IHC) was purchased from Proteintech Group (USA). Hoechst 33342 and LysoTracker^®^ Green DND26 were purchased from Thermo Fisher Scientific (USA). Anti-NAPDH antibody was purchased from Bioworld Technology (Nanjing, China). DMEM medium, fetal bovine serum and 0.25% trypsin solution were purchased from Gibco (USA). ELISA kits for proteins, urea nitrogen and creatinine detection were purchased from Jiancheng Bioengineering Institute (Nanjing, China). The other materials such as dimethylformamide (DMF), dichloromethane (DCM) and triethylamine (TEA) were of analytical grade.

### Cell culture and animals

A proximal tubule epithelial cell line from human kidney (HK-2) was obtained from the Shanghai Institutes for Biological Sciences of Chinese Academy of Sciences. HK-2 cells were cultured in DMEM medium (Gibco, USA) containing 10% fetal bovine serum and 100 U/mL penicillin/streptomycin and were maintained in a humidified atmosphere of 5% CO_2_ at 37 °C. Rabbit red blood cells (RBC) were provided by the Laboratory Animal Center at Zhejiang Chinese Medical University.

Male C57BL/6 mice (also known as C57BL/6J) were supplied by the Laboratory Animal Center of Zhejiang Chinese Medical University; the animals were maintained at 25 °C and 45% relative humidity and were exposed to 12 h light/12 h dark cycles. All experiments were performed in accordance with the guidelines for the care and use of animals established by the Zhejiang Chinese Medical University. The animal experiments (License No. 2016063) were approved by the Institutional Animal Ethics Committee of Zhejiang Chinese Medical University.

### Synthesis of PCL-PEI

PEI was synthesized by cationic ring-opening polymerization 24, 25. Briefly, 2-methyl-2-oxazoline (2 mL, 23.5 mmol) and methyl p-toluenesulfonate (0.186 g, 1 mmol) were dissolved in dry acetonitrile (10 mL), followed by bubbling with nitrogen to remove the dissolved oxygen. After stirring for 24 h at 75 °C, ethylenediamine (0.5 mL, 8 mmol) was added and stirred for another 6 h. Acetylated polyethyleneimine (APEI) was obtained by precipitation in cold diethyl ether thrice. APEI was then dissolved in hydrochloric acid (100 mL, 15%, mass fraction) and refluxed for 12 h at 110 °C to remove the acetyl. The pH was adjusted to 8.5 with a NaOH solution after cooling. The solution was concentrated and then dialyzed (Spectra/Por-6 dialysis tubing was made of regenerated cellulose, type: Mw cut-off 1 kDa, Spectrum) against water for 24 h by replacing the fresh dialysis buffer every 8 h (volume ratio of sample to dialysis buffer was 1/100) and freeze-dried PEI was obtained with a 26.6% yield (about 0.53 g). The structure was confirmed by proton nuclear magnetic resonance (^1^H NMR) (Avance-400 NMR, Bruker). ^1^H NMR of PEI (D_2_O, 400Hz, *δ*): 3.46 (C***H_3_***NHCH_2_-, 3.0H), 2.95 (-NHC***H_2_***C***H_2_***-, 83.6H). The theoretical molecular weight calculated from the ^1^H NMR was 0.9 kDa (83.6/4=21, 21 repeating units in PEI. The molecular weight of each repeating unit was 43; therefore, the theoretical molecular weight was 0.903 kDa). The molecular weight was determined by gel permeation chromatograph (GPC) (Waters 1515, Waters Co., USA) and was about 0.82 kDa, which matched with the theoretical molecular weight. The peak at 1.85 ppm in ^1^H NMR of PEI was the remaining C***H_3_***CO-. There was about 1 C***H_3_***CO- in every 21 repeating units of PEI. The removal rate was greater than 95%, indicating that the polymers met the purity requirements of the compound.

Carboxyl terminated PCL (0.37 g, 0.1 mmol), DCC (0.042 g, 0.2 mmol) and NHS (0.023 g, 0.2 mmol) were mixed in acetonitrile (10 mL) and stirred at room temperature for 4 h, followed by adding PEI (0.11 g, 1.2 mmol) and stirring at 45 °C for 12 h. The solution was dialyzed (dialysis tubing was made of regenerated cellulose, type: MEMBRA-CEL MD44, Mw cut-off 3.5 kDa, Viskase) against methanol for 24 h by replacing the fresh dialysis buffer every 8 h (volume ratio of sample to dialysis buffer was 1/100) and dried to obtain PCL-PEI (about 0.41 g, 89.2% yield). ^1^H NMR of PCL-PEI (CD_3_OD, 400Hz, *δ*): 1.40 (-COCH_2_CH_2_C***H_2_***CH_2_CH_2_O-, 73.8H), 1.63 (-COCH_2_C***H_2_***CH_2_C***H_2_***CH_2_O-, 122.1H), 2.30 (-COC***H_2_***-, 63.1H), 2.91 (-NHC***H_2_***C***H_2_***-, 60.3H), 4.06 (-C***H_2_***O-, 64.0H). The theoretical molecular weight calculated by ^1^H NMR was 4.35 kDa, and the molecular weight of PCL-PEI was about 4.6 kDa determined by GPC.

### Synthesis of fluorescence-labeled PCL-PEI

PCL-PEI (100 mg) and Cy5-NHS (0.5 mg) were dissolved in DMF (5 mL) and stirred overnight in the dark. The solution was purified by dialysis (dialysis tubing was made of regenerated cellulose, type: MEMBRA-CEL MD44, Mw cut-off 3.5 kDa, Viskase) in methanol for 24 h by replacing the fresh dialysis buffer every 8 h (volume ratio of sample to dialysis buffer was 1/100) and dried to obtain fluorescence-labeled PCL-PEI. The grafting ratio of the Cy5-labeled PCL-PEI (PCL-PEI^Cy5^) was about 2.2% and was detected by fluorescence intensity using a microplate reader (SpectraMax M_5_, Molecular Devices).

### Synthesis of DSPE-PEG-KTP

DSPE-PEG-NHS (150 mg, 0.05 mmol) and KTP (82 mg, 0.1 mmol) were dissolved in DMF (5 mL) and stirred for 12 h at 45 °C. DSPE-PEG-KTP was obtained (about 144 mg, 75.4% yield) by dialysis (dialysis tubing was made of regenerated cellulose, type: MEMBRA-CEL MD44, Mw cut-off 3.5 kDa, Viskase) against methanol for 24 h by replacing the fresh dialysis buffer two times (ratio of sample to dialysis buffer was 1/100) and freeze drying. The coupling purity was verified by matrix-assisted laser desorption ionization time-of-flight mass spectrometry (MALDI-TOF-MS). The analytical sample was prepared by mixing the sample solution (1 µL, 5 mg/mL dissolved in methanol) with the matrix solution of 2,5-dihydroxybenzoic acid (1 µL, 10 mg/mL dissolved in the mixture of the water and acetonitrile) on the stainless-steel probe, and this mixture was allowed to dry at room temperature. The laser power was chosen in the range of 50 ~ 100 µJ.

### Critical micelle concentration (CMC) of PCL-PEI

CMC was determined by the pyrene method 26. Briefly, pyrene (100 µL, 20 µg/mL in acetone) was placed into a centrifuge tube and acetone was evaporated. The polymer suspension (1 mL) was added to the solution at final pyrene concentration of 2 µg/mL. After tubes were stirred at room temperature overnight in the dark, the fluorescence excitation spectrum was measured at the wavelengths of 300 ~ 360 nm (emission wavelength at 390 nm). The ratio of fluorescence intensities at the excitation wavelengths of 338 nm and 333 nm (I_338_/I_333_) was calculated and CMC was determined from the fitted curves.

### HPLC assay of RH

High performance liquid chromatography (HPLC) (Agilent-l200) was conducted using a Platisil ODS column (150 × 4.6 mm, 5 µm) at 25 °C. The mobile phase was eluted at 1.0 mL/min and consisted of methanol/0.1% phosphoric acid (75:25, v/v). The detector was monitored at 254 nm. Fitted curves of peak areas (*A*)-RH concentrations (*C*) fitting curves were obtained. The intra- and inter-day precision was evaluated by testing the RH samples (1, 10 and 40 µg/mL) 5 times daily for 3 consecutive days. The accuracy was obtained by determining the recovery of drug from the excipients at 3 quantities of RH (80%, 100% and 120% w/w) in the prescriptions.

### Preparation and characterization of liponanoparticles

The KLPPR were prepared by nanoprecipitation and film dispersion method. Briefly, RH (2 mg) and PCL-PEI (30 mg) were dissolved in a mixture solvent (3 mL, acetonitrile/methanol, 2/1 v/v). The mixture was added dropwise to deionized water (10 mL, pH = 6) under agitation (1000 r/min) and stirred for 20 min. The organic solvent was evaporated under reduced pressure to obtain PPR. CHEMS (0.98 mg, 2 µM), DOPE (4.46 mg, 6 µM), DSPE-PEG (5.6 mg, 2 µM), and DSPE-PEG-KTP (3.7 mg, 1 µM) were dissolved in chloroform (1 mL) and dispersed ultrasonically. Then the solution was placed in a flask and evaporated under reduced pressure to form a lipid membrane, followed by adding PPR suspension and ultrasonic rehydration to form the KLPPR liponanoparticles.

A small fraction of the polymer was conjugated with a pair of fluorescence resonance energy transfer (FRET) dyes, Cy5 and Cy5.5, and used to label PCL-PEI and DSPE-PEG respectively. PCL-PEI^Cy5^ and DSPE-PEG^Cy5.5^ were used to fabricate FRET liponanoparticles (KLPPR^FRET^) whose FRET efficiency was correlated to the percentage of PPR coated in lipid shell, the KLPPR degree of stability. The Cy5-labeled KLPPR (KLPP^Cy5^R), LPPR (without DSPE-PEG-KTP), Cy5-labeled LPPR (LPP_Cy5_R) were prepared by the same method.

The fluorescence spectra of KLPPR^FRET^ were measured using a fluorescence spectrometer at an excitation wavelength of 640 nm with a slit width of 2 nm, integration time of 0.1 s and increment of 0.5 nm. Emission spectra were collected from 600 to 750 nm. The FRET ratio (R) was calculated from the intensities of the fluorescence at 670 nm and 710 nm.

The average size and zeta potential of PPR, liposomal coatings and KLPPR were measured using a dynamic light scattering analyzer (Nano-ZS 90, Malvern) after proper dilution with distilled water. The refractive index of the nanoparticle was chosen and set at 1.59 to determine the size by volume percent (volume %). The stability of size and zeta potential of KLPPR were monitored over 48 h. The morphology was characterized by transmission electron microscopy (TEM) (JEM-1200EX, JEOL) on the copper grid. KLPPR suspension (1 mL) was centrifuged at 20,000 r/min (rotor type: JA-21, Avanti JXN-30, Beckman) for 30 min and the concentration of free RH in supernatant was determined by HPLC. The encapsulation efficiency (EE) and drug-loading efficiency (LE) were calculated using equations (1) and (2), respectively:


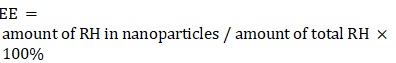
 (1)


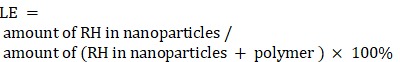
 (2)

### *In vitro* drug release

The drug release profiles were investigated with the dialysis method in a phosphate-buffered saline (PBS) solution (pH 7.4, containing 2% Tween 80 and 1% glycerol). KLPPR, PPR, and RH solution (RH-sol) were placed into dialysis bag (dialysis tubing was made of regenerated cellulose, type: MEMBRA-CEL MD44, Mw cut-off 3.5 kDa, Viskase) and were immersed into 500 mL medium in a 37 °C shaker (60 r/min). Samples (1 mL) were collected at different times (0.25, 0.5, 1, 2, 3, 4, 6, 8, 12, 24, and 48 h) and the mixture was replenished with an equal volume of fresh medium. RH content was quantified by HPLC and the cumulative release rate (Q) was calculated using equation (3).


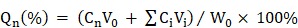
 (3)

where Q_n_, C_n_, V_0_, V_i_, C_i_, and W_0_ refer to the percentage of the cumulative release at each collection time, the drug concentration at t_n_, the released medium volume, the removed sample volume, the drug concentration at t_i_, and the original total drug weight, respectively.

### Hemolysis test

The erythrocytes were isolated from anticoagulated fresh rabbit blood by centrifugation at 2000 r/min for 5 min and washed with PBS solution (pH 7.4) 3 times. KLPPR and PPR suspension were added to centrifuge tubes and diluted with PBS solution to obtain a final volume of 0.9 mL with varying concentrations. A stocking solution of erythrocytes (10^8^ cells/mL in PBS solution) was prepared and 0.1 mL was added to the dilutions. After incubation in 37 °C shaker (60 r/min) for 2 h, intact red blood cells were obtained by centrifuging at 2000 r/min for 5 min. The absorbance (A) of the supernatant at 540 nm was measured. Hemolysis ratios were obtained using equation (4), where the PBS solution and water were taken as negative and positive control, respectively.

Hemolysis ratio = (A_sample_-A_negative_) / (A_positive_-A_negative_) × 100% (4)

### Cytotoxicity assay

HK-2 cells were digested with 0.25% trypsin, collected, centrifuged, and resuspended using DMEM medium. The cells (5×10^4^ cells/mL 180 µL) were placed into 96-well plates and the nanoparticles suspensions (20 µL) were added 12 h later; PBS was used as control. Each test was performed six repetitions. After 48 h, MTT solution (5 mg/mL, 20 µL) was added and incubated for additional 4 h. The medium was replaced with dimethyl sulfoxide (200 µL) and the A of the solution at 570 nm and 620 nm was measured. The cell viability was calculated using equation (5).

Cell viability = (A_570 nm_-A_620 nm_) _Sample_ / (A_570 nm_-A_620 nm_) _Control_ × 100% (5)

### Cellular uptake

HK-2 cells (1×10^5^ cells/mL, 1 mL) were seeded in 12-well plates and cultured for 12 h. KLPP^Cy5^R, LPP^Cy5^R and PP^Cy5^R nanoparticles (100 µL) with equal fluorescence intensity were added and incubated for 0, 0.25, 0.5, 1, 1.5 2, and 3 h. The medium was removed, and the cells were rinsed with PBS three times, digested, harvested and analyzed using flow cytometry to determine the average fluorescence intensity of HK-2 cells at different incubation times. The uptake rate curves were determined to evaluate the uptake efficiency. To investigate the effect of KTP on uptake efficiency, after the preincubation of the solutions with different concentrations of KTP for 1 h, KLPP^Cy5^R, LPP^Cy5^R, and PP^Cy5^R nanoparticles (100 μL) with equal fluorescence intensity were added and incubated for 0.5 h. Cells were tested as mentioned above.

### Mechanism of endocytosis

HK-2 cells (1×10^5^ cells/well, 1 mL) were cultured in 12-well plates for 12 h. Chlorpromazine (50 µM, clathrin-mediated endocytosis inhibitor), filipin III (7.5 µM, caveolae-mediated endocytosis inhibitor), wortmannin (5 µM, phosphatidylinositol 3-kinases-mediated macropinocytosis inhibitor), and cytochalasin D (5 µM, actin-polymerization inhibitor) were added to the medium for 2 h incubation, respectively 27, 28. Another group of cells was also cultured at 4 °C for 2 h. Then, KLPP^Cy5^R, LPP^Cy5^R, and PP^Cy5^R nanoparticles with equal fluorescence intensity (100 μL) were added and incubated for 2 h, respectively. Medium was discarded, and cells were washed with heparin sodium solution (2 mg/mL) once, digested with 0.25% trypsin (0.02% EDTA) solution, collected into centrifugal tube, centrifuged, washed with PBS once, and suspended in PBS (0.5 mL). The positive cells rates were detected by flow cytometry.

### Subcellular distribution

HK-2 cells (1×10^5^ cells/dish, 1 mL) were cultured in a confocal dish for 24 h. The lysosomes were labeled with LysoTracker^®^ Green DND26 (0.2 µL) for 30 min and the nuclei were stained with Hoechest33342 (2 drops) for 20 min. The cells were washed with PBS once and 1 mL fresh serum-free DMEM medium was added containing KLPP^Cy5^R and PP^Cy5^R nanoparticles (10 µL). The mixtures were incubated for 15 min, 1 h and 3 h. The subcellular distribution images of nanoparticles were photographed with a confocal laser scanning microscope (CLSM) (Nikon A1, Nikon Corporation, Japan) using 405, 488 and 640 nm wavelength channels.

### DN model mice

The experimental protocol of DN model mice was described by the Animal Models of Diabetic Complication Consortium 29 and the Animal Models of Disease 30, 31. Male C57BL/6 mice were fasted overnight and intraperitoneally injected with STZ (200 mg/kg, dissolved in 50 mM pH 4.5 citrate buffer solution); the mice in the healthy control group (HC) were injected intraperitoneally with a citrate buffer. The mice were fed water containing 10% sucrose for 3 days after the STZ injection. A week later, mice were fasted overnight and the fasting blood glucose (FBG) were tested by using a One Touch Basic Blood Glucose Monitoring Apparatus. Diabetes was defined as FBG ≥ 15 mmol/L. Body weight and FBG of the mice were monitored for 5 weeks. The worsening of diabetes symptoms or the occurrence of serious complications was a sign of diabetes developing into early DN at 5 weeks after STZ treatment 29-31.

### *In vivo* stability

The abdomen of mice was depilated and the skin along with the abdominal midline was fixed on a microscope slide using Histoacryl (3M VetbondTM, USA). Under the LSM710NLO confocal microscope, one clear blood vessel was identified. The KLPPR^FRET^ solution in PBS (200 µL, equivalent to 5 mg/kg RH) was injected into DN model mice via the tail vein. At timed intervals, the 600-750 nm fluorescence spectra of the blood excited with a 633-nm HeNe laser were recorded and the corresponding images were taken from the 710 nm fluorescence channel. The concentration of the KLPPR^FRET^ remaining in the blood was calculated from the FRET fluorescence and their FRET ratio was calculated from the spectra to measure *in vivo* stability.

### The blood clearance in DN model mice

The DN mice were intravenously injected with RH-sol and KLPPR (dose equivalent to RH 5 mg/kg, 4 mice in each group). Blood samples (50 µL) were collected via the orbital venous plexus of mice at timed intervals (2 min, 15 min, 30 min, 1 h, 2 h, 3 h, 4h, 6h, 8h, 12h), and then mixed with heparin solution (1 mg/mL, 50 µL). At the end of experiments, the mice were sacrificed and the kidneys were excised, weighed and ground by the TissueLyser®-LT (Qiagen, Germany) for calculating the concentration of RH in kidney. The samples were separated from the blood or tissue homogenates by centrifuge at 5,000 rpm for 5 min at 4 °C. 50 µL of samples were diluted with acetonitrile (950 µL) to free and extract RH. The obtained mixtures were thoroughly vortexed and ultrasonicated, and then centrifuged at 5000 rpm for 5 min. 500 µL of supernatants was removed and concentrated for HPLC analysis. The RH content was calculated according to the standard curve and pharmacokinetic parameters were analyzed by accordingly.

### Biodistribution in DN model mice

The DN mice (n = 4) were intravenously injected with LPP^Cy5^R and KLPP^Cy5^R (200 µL/20 g). The chest and abdomen of mice were depilated and urine was collected in metabolic cage. The real-time distribution of nanoparticles was observed and quantitively analyzed via an *in vivo* imaging system (IVIS Lumina II, PerkinElmer) at 8 h. The mice were sacrificed and the major organs (hearts, livers, spleens, lungs, kidneys, and intestines) were excised, washed with saline, weighed, and imaged. The total fluorescence intensity of the organs and urine were recorded by the IVIS Lumina II.

### Pharmacodynamics study

The DN mice were injected with Lantus (subcutaneously, 0.5 U/mouse), RH-sol, LPPR and KLPPR (intravenously, dose equivalent to RH 5 mg/kg, 6 mice in each group). The HC group and DN control group were treated intravenously with saline (6 mice in each group). The administration was performed every other day for 20 days. The FBG and body weight of mice were measured and recorded every other day.

At the end of the treatment, the mice were kept in metabolic cages and the 24-hour urinary volume (24h-UV) was collected and 24-hour urinary protein excretion (24h-UPE) and urinary creatinine (UCr) were measured. Whole blood samples were collected, centrifuged and analyzed to measure serum urea nitrogen (SUN), serum creatinine (SCr), and creatinine clearance rate (CCr). The mice were sacrificed and kidneys were dissected and weighed. The ratios of kidney weight and body weight (kidney index) were calculated. One kidney was fixed in 4% paraformaldehyde and embedded in paraffin for hematoxylin and eosin staining (H&E) and periodic acid-schiff (PAS) staining to observe the renal lesions under an optical microscope and evaluate the pathological morphology quantitatively. The expression of fibronectin (FN) in kidney was analyzed by immunohistochemistry (IHC). The other kidney was stored at -80 ℃ and was homogenized for proteins extraction. The expression of TGF-β1 and Smad2/3 associated with advanced kidney fibrosis were analyzed via Western blotting. The CCr was calculated using equation (6).


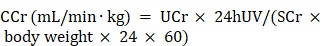
 (6)

### Data analysis

Each measurement was at least performed in triplicates. The data were expressed as mean ± standard deviation (SD). The significant differences in the mean values were assessed by one-way analysis of variance (ANOVA). A statistical test with a *P* < 0.05 was considered statistically significant.

## Results and Discussion

### Synthesis and characterization of polymers

**Figure 1:**
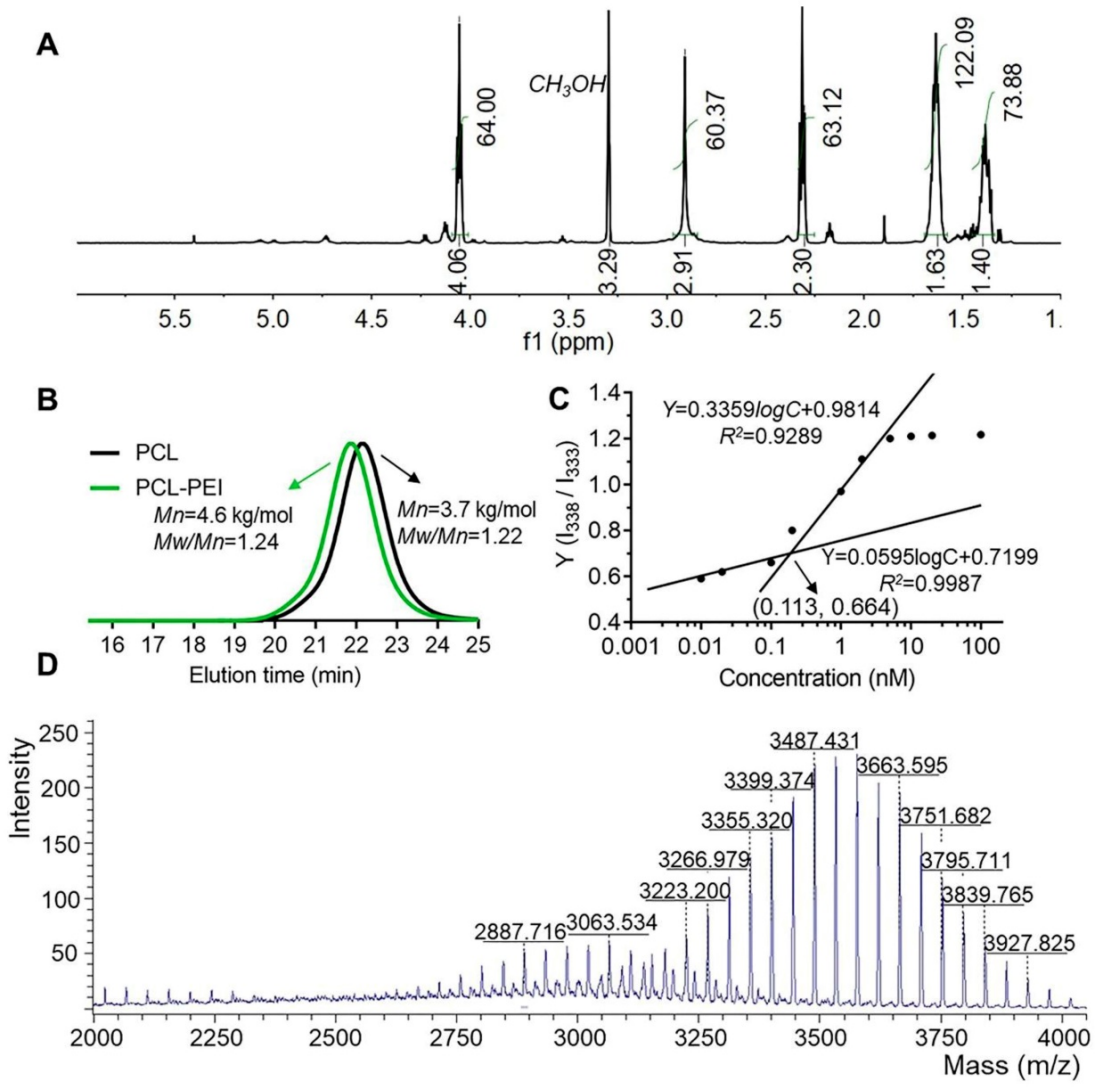
** Characteristics of the PCL-PEI polymer and DSPE-PEG-KTP.** ^1^H NMR spectrum (A) and GPC spectra of the polymers (B). I_338_/I_333_ ratio shown in fluorescence intensity as a function of the concentration and fitted curves obtained by logarithm model (C). The MALDI-TOF-MS spectrum of DSPE-PEG-KTP (D).

The synthetic route for the PCL-PEI is presented in Figure S1A. APEI was first synthesized from cationic open-loop polymerization of 2-methyl-2-oxazoline. PCL-PEI was then synthesized via the amidation reaction between the carboxyl terminated PCL and PEI, the latter was obtained by removing the acetyl group under hydrochloric acid reflux, and their structures and purity were confirmed by ^1^H NMR (Figure 1A and S1B). ^1^H NMR of PEI (D_2_O, 400Hz, *δ*): 3.46(C***H_3_***NHCH_2_-, 3.0H), 2.95(-NHC***H_2_***C***H_2_***-, 83.6H). The peak at 1.85 ppm represented the remaining C***H_3_***CO-, and there was about 1 C***H_3_***CO- in every 21 repeating units of PEI. The removal ratio of the acetyl group was greater than 95%, confirming that the polymers met the purity requirements of the compound. ^1^H NMR of PCL-PEI (CD_3_OD, 400Hz, *δ*): 1.40(-COCH_2_CH_2_C***H_2_***CH_2_CH_2_O-, 73.8H), 1.63(-COCH_2_C***H_2_***CH_2_C***H_2_***CH_2_O-, 122.1H), 2.30(-COC***H_2_***-, 63.1H), 2.91(-NHC***H_2_***C***H_2_***-, 60.3H), 4.06(-C***H_2_***O-, 64.0H). The molecular weight was about 4.6 kDa determined by GPC (Figure 1B); the results agreed well with the theoretical molecular weight. The GPC traces exhibited a narrow and uniform distribution with a polydispersity index (PDI) of 1.24. In general, these results verified the successful synthesis of PCL-PEI. PCL-PEI was labeled by Cy5 with a grafting ratio of 2.2% (mole ratio) as determined by the fluorescence spectrum intensity (Figure S2A). The CMC of PCL-PEI self-assembly micelles was determined by pyrene method. The values of I_338_/I_333_ were calculated and curves were fitted; the CMC value was considered the inflection point of the plot 26. As shown in Figure S2B and Figure 1C, as the polymers concentrations increased, the excitation light intensity of the pyrene increased rapidly and a red-shift of excitation wavelength from 333 nm to 338 nm was observed. The CMC of PCL-PEI was 0.113 nmol/L, which was lower than that of the PEG-PCL with the same molecular weight mainly because of the weaker hydrophilicity of the PEI than the PEG 32.

The highly efficient coupled reaction between the active ester-modified lipid and amino groups of peptides has been widely utilized for peptide and antibody modification 33. DSPE-PEG-KTP was obtained (about 144 mg, 75.4% yield) by coupling DSPE-PEG-*NHS* and *NH_2_*-KCSAVPLC-COOH. Since the m/z values of commercialized DSPE-PEG-*NHS* and *NH_2_*-KCSAVPLC-COOH were about 2300~3300 m/z and 820 m/z respectively and the removed N-hydroxysuccinimide ester was 115 m/z, the DSPE-PEG-KTP was theoretically in the range of 3000~3900 m/z (Figure 1D). The ^1^H NMR spectra (Figure S3) and MALDI-TOF-MS spectrum (Figure S4) supported the successful synthesis and characterization of the DSPE-PEG-KTP.

### HPLC assay of RH

The RH was detected by HPLC to assess the specificity, stability, and repeatability. HPLC could detach and detect the RH with specific chromatograms even in the plasma (Figure S5). The peak areas (*A*)-RH concentrations (*C*) of the standard curve were fitted with a linear relationship in the range of 0.1 ~ 40 µg/mL (regression equation: *A*=71.272•*C*-12.817, *R^2^*=0.9998). Relative standard deviation values of the intra- and inter-day precision were within the acceptable variable limits of variation (0.54%, 0.47%, 0.58% and 1.05%, 0.86%, 0.72% for 1, 10 and 40 μg/mL RH, respectively) and all results were less than 2%. The recovery rates of the RH samples (80%, 100%, and 120% w/w) were 96.63 ± 2.38%, 97.01 ± 0.75%, and 97.27 ± 1.56%, respectively. Hence, due to satisfactory precision and accuracy, the HPLC method met the methodological requirements.

### Preparation and characterization of nanoparticles

**Figure 2:**
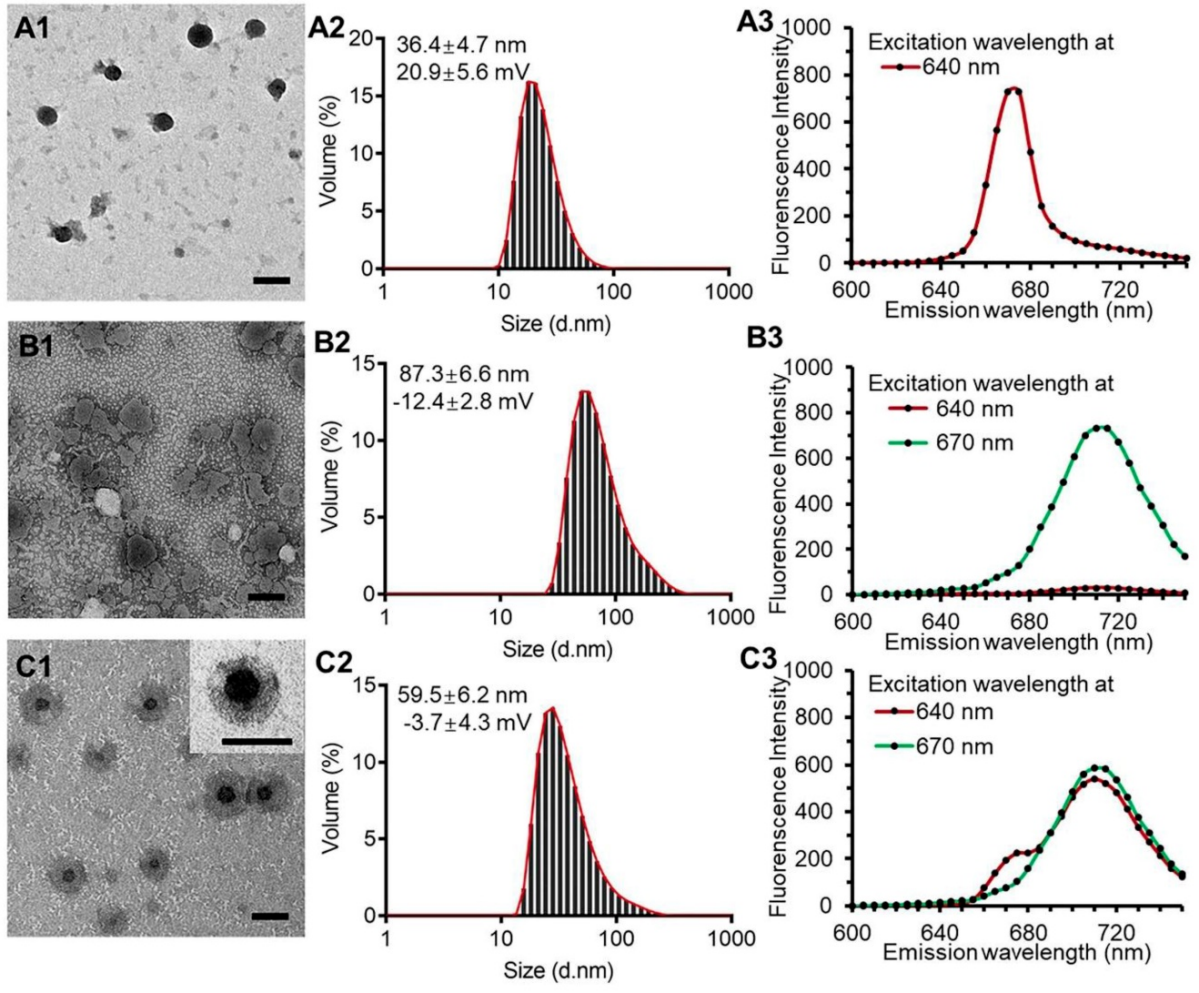
** TEM images, particle size distribution and dye-labeled fluorescence spectra.** PPR nanoparticles (A1, A2, A3), blank liposomal coatings (B1, B2, B3), and KLPPR liponanoparticles (C1, C2, C3) with a yolk-shell structure. The scale bar is 50 nm.

The PPR obtained nanoparticles had monodispersed spherical structure with no appreciable adhesions and aggregations in TEM images and had a diameter of 36.4 ± 4.7 nm with the polydispersity index of 0.13 and a zeta potential of 20.9 ± 5.6 mV (Figure 2, A1 and A2). The blank liposomal coatings had a particle size of 87.3 ± 6.6 nm with irregular shape and a zeta potential of -12.4 ± 2.8 mV (Figure 2, B1 and B2). The KLPPR were assembled by electrostatic interaction of the oppositely charged PPR and lipid, and had a yolk-shell structure with uniform size of 59.5 ± 6.2 nm and a zeta potential of -3.7 ± 4.3 mV (Figure 2, C1 and C2). Cy5 (Ex: 640 nm; Em: 670 nm) and Cy5.5 (Ex: 670 nm; Em: 710 nm) were selected as a FRET pair and separately conjugated to the PCL-PEI and DSPE-PEG to prevent dye release from the nanoparticles, which was always found in physical encapsulation. Once excited at 640 nm, PPR^Cy5^ had a strong excitation wavelength around at 670 nm (Figure 2A3), while the liposomal coatings^Cy5.5^ could be excited at 670 nm rather than 640 nm (Figure 2B3). When the liposomal coatings coated onto the PPR, the KLPPR^FRET^ had strong Cy5.5 fluorescence at 710 nm with weak Cy5 fluorescence at 670 nm, indicating a strong FRET occurred and a core-shell structure formed.

**Figure 3:**
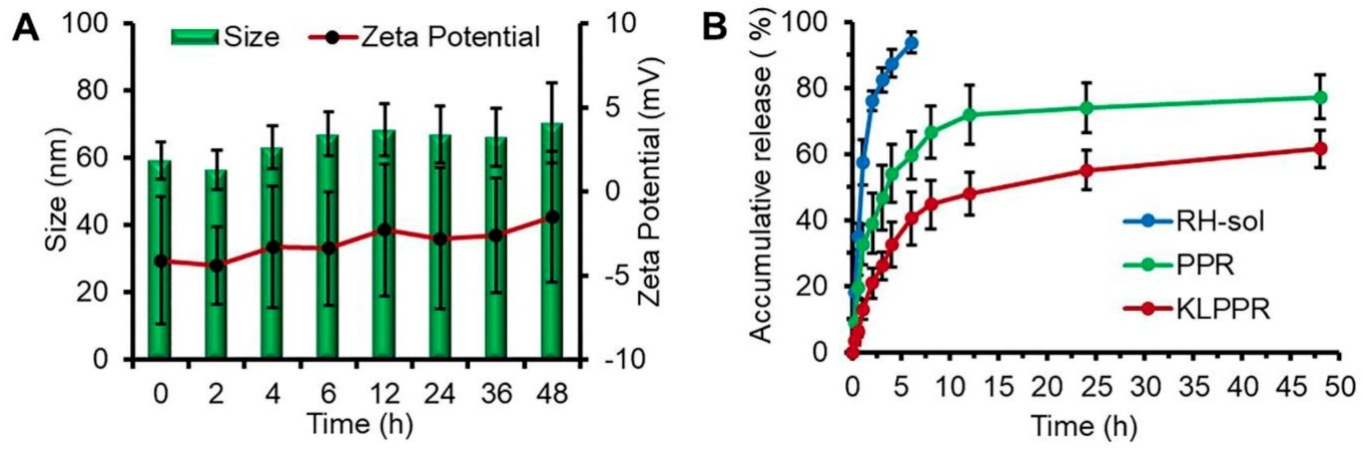
***In vitro* stability and drug release properties.** Changes of size and zeta potential of KLPPR within 48 h (A). Drug release profiles of RH-sol, PPR, and KLPPR in PBS (pH 7.4, containing 2% Tween 80 and 1% glycerol) (B).

Size and zeta potential of KLPPR could maintain stable within 48 h with no significant changes (Figure 3A), suggesting their good pharmaceutical stability. The KLPPR had an EE of 90.22 ± 4.26% and LE of 5.17 ± 0.69%, indicating that the PEI moiety in the polymer enhanced the self-assembly ability and improved the loading capability for RH due to ionic interaction between carboxylic groups or phenol hydroxyl groups in RH and the repeated amine group units in PEI. The other liponanoparticular formulations of KLPP^Cy5^R, KLPPR^FRET^, LPPR and LPP^Cy5^R possessed a particle size of 60 ± 10 nm with PDI of 0.1 ~ 0.2 and zeta potential of -5.4 ± 4.7 mV, which was consistent with that of the KLPPR.

### Characteristics of *in vitro* drug release

The release profiles of RH-sol, PPR, and KLPPR are shown in Figure 3B. RH released fast in the RH-sol group, and the Q was close to 90% within 4 h. The PPR had a burst release within 3 h with a Q of 40%, and a subsequently sustained release with a Q of 74.3% at 48 h. In contrast, the KLPPR showed sustained release behavior without a significant burst release with a Q of about 25% and 61.8% at 3 h and 48 h, respectively. Aside from the physically embedded RH driven by hydrophobic action in the PPR nanoparticles, the RH was adsorbed on the outer layer of the nanoparticles via ionic interaction between the amino group of PEI and the carboxyl group and phenol hydroxyl group of RH, which resulted in the burst release. When the PPR nanoparticles were further encapsulated by liposomal coatings, the undesirable burst drug release was prevented. The release kinetics of KLPPR were predicted by the classically zero-order, first-order, and Higuchi model (Table S1) 34. The drug release curve of KLPPR in PBS was fitted well with the Higuchi model equation (Q = 0.979t^1/2^ + 0.0648, *R^2^*= 0.8655), proving the sustained-release of RH.

### Hemolysis and cytotoxicity assay

**Figure 4:**
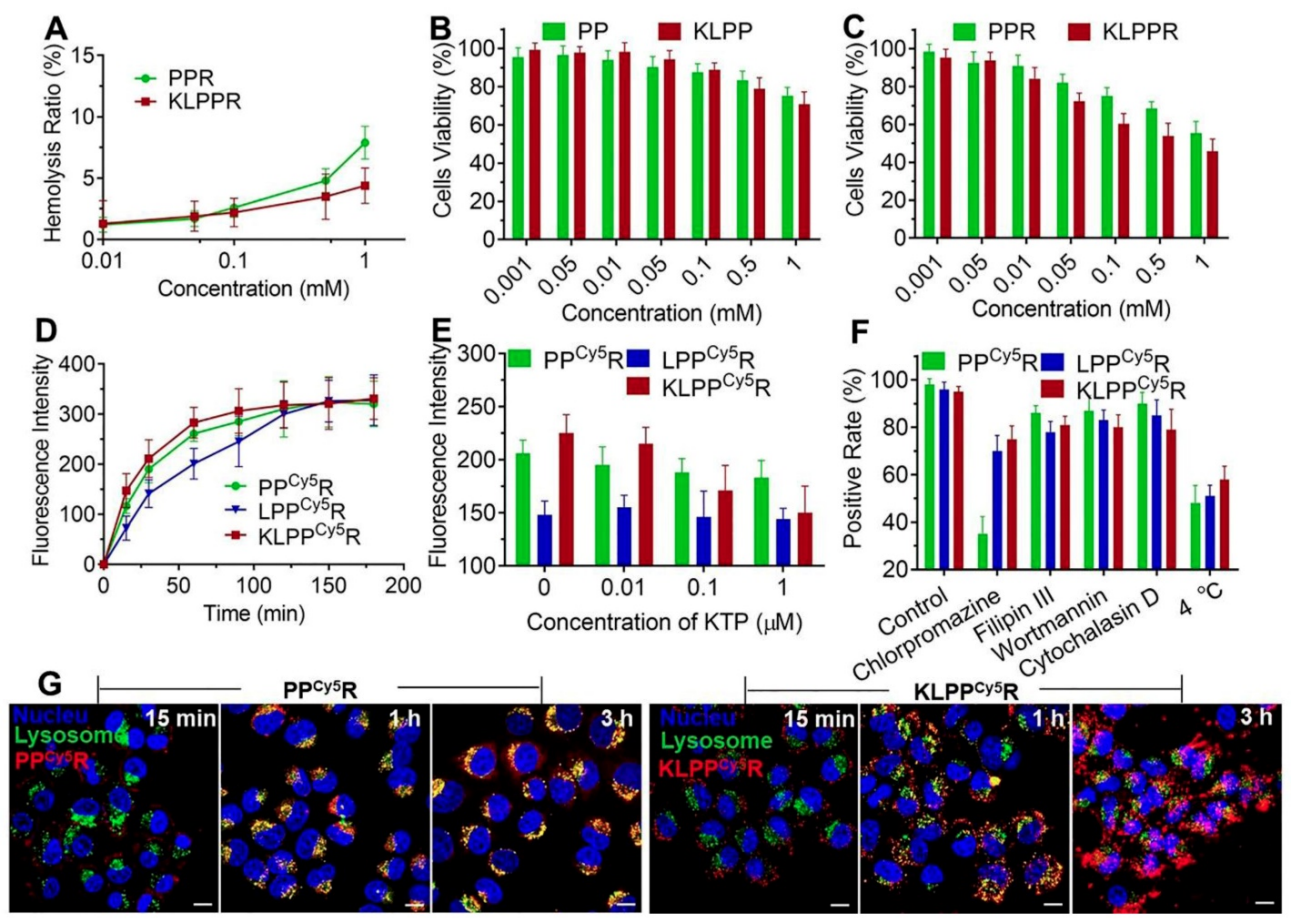
** Biocompatibility, cytotoxicity, cellular uptake and subcellular distribution of nanoparticles.** Hemolysis assay (A) was conducted using rabbit red blood cells. *In vitro* cytotoxicity on HK-2 cells of blank nanoparticles (B) and drug-loaded nanoparticles (C) were measured with the MTT assay. Changes in the cellular uptake efficiency with interval time (D), the different free KTP concentrations (E) and the endocytosis inhibitors and 4 °C (F) on cellular uptake efficiency were determined using flow cytometer. Subcellular distribution of PP^Cy5^R and KLPP^Cy5^R (G). HK-2 cells were imaged with a CLSM and the nuclei were stained with Hoechst 33342 (blue), the lysosomes were labeled with LysoTracker^®^ Green DND26 (green) and the nanoparticles were labeled with Cy5 (red). The scale bar is 50 μm.

The biocompatibility and cytotoxic responses of PPR and KLPPR were evaluated using hemolysis assay and MTT test. PPR exhibited higher hemolytic activity than KLPPR and both elicited hemolysis with an increase in the polymer concentration. The values were less than 5% at concentrations of less than 0.5 mmol/L, indicating no erythrocyte-associated detrimental effects (Figure 4A). MTT assay was further used to examine the cytotoxicity of the blank and drug-loaded nanoparticles (Figure 4, B and 4C). The cell viability remained above 90% even at a high polymer concentration (0.5 mmol/L), demonstrating the good biocompatibility of the blank nanoparticles. KLPPR exhibited dose-dependent behavior with regard to the HK-2 cells and showed higher cytotoxicity than that of PPR, which was mainly attributed to the enhanced cellular uptake of KLPPR and RH's pharmacological effects of inhibiting cell proliferation 35, 36.

### Mechanism of cellular uptake

The cellular uptake of nanoparticles at different time was further examined using flow cytometry. As showed in Figure 4D, the fluorescence intensity of cells increased with increasing incubation time. The LPP^Cy5^R exhibited slower uptake than the KLPP^Cy5^R and PP^Cy5^R and were internalized fully at 120 min. In contrast, more than 60% of the KLPP^Cy5^R and PP^Cy5^R were internalized within the first 30 min and the cellular uptake was complete after 60 min. The higher efficiency of the PP^Cy5^R was attributed to the adherence interaction between the negatively charged proteins or phospholipids in cell membrane and the positively charged nanoparticles. A positive charge would bring about concerns of proteins absorbance in physiological environment and instability of nanoparticles, which would cause their rapid clearance by the reticuloendothelial system and unproductive intravenous administration 37, 38. In contrast, the negatively charged KLPP^Cy5^R were able to bind specifically and actively to the HK-2 cells due to KTP mediation, which resulted in higher cellular uptake efficiency than that of the LPP^Cy5^R.

Subsequently, the effect of the KTP concentration on the cellular uptake efficiency was examined and the results are illustrated in Figure 4E. The cellular uptake efficiency of KLPP^Cy5^R decreased with increasing of KTP concentration in the culture medium. At 1 μmol/L KTP, the cellular uptake efficiency of the KLPP^Cy5^R was the same as that of LPP^Cy5^R, but the KTP had no effect on LPP^Cy5^R and PP^Cy5^R nanoparticles, verifying the KTP-induced enhancement of the renal cellular uptake of KLPP^Cy5^R.

In order to investigate the internalization pathway of KLPP^Cy5^R, cells were preincubated at different temperatures (4 and 37 °C) and in the presence of different endocytic inhibitors e.g. chlorpromazine (50 µM, an inhibitor of clathrin-mediated endocytosis), filipin III (7.5 µM, an inhibitor of caveolae-mediated endocytosis), wortmannin (5 µM, an inhibitor of macropinocytosis) and cytochalasin D (5 µM, an inhibitor of actin-dependent endocytosis) (Figure 4F). Flow cytometry results of PP^Cy5^R showed that chlorpromazine and low temperature induced positive cells rate reduction to 35% and 48% from 95%, respectively, but filipin III, wortmannin, and cytochalasin D had little influence on the internalization efficiency; the positive cells rate was higher than 85%, indicating the energy-dependent clathrin-mediated endocytosis of PP^Cy5^R. The cellular uptake efficiency was significantly lower at 4 °C than at 37 °C for the KLPP^Cy5^R and LPP^Cy5^R (51% and 58%* vs.*96% and 95%, respectively), and was slightly suppressed by preincubation with chlorpromazine (70% and 74% for KLPP^Cy5^R and LPP^Cy5^R, respectively), but was not obviously influenced by the other three inhibitors (more than 80% for KLPP^Cy5^R and LPP^Cy5^R). Considering the fact that the envelopment of lipid coatings changed the internalization pathway 28, 39, multiple pathways including clathrin-mediated endocytosis, caveolae-mediated endocytosis and membrane fusion were speculated to participate in the internalization of KLPP^Cy5^R and LPP^Cy5^R. Therefore, the non-lysosomal pathway of membrane fusion was likely the dominant internalization pathway.

### Subcellular distribution

The subcellular distribution of PP^Cy5^R and KLPP^Cy5^R was determined with CLSM (Figure 4G and Figure S6). The PP^Cy5^R and KLPP^Cy5^R first interacted with the cells for 15 min and for the most part was internalized into the cell after 1 h incubation; this was likely due to the binding of electropositive PP^Cy5^R to the cell membrane and the mediation of KTP in KLPP^Cy5^R. The PP^Cy5^R with red fluorescence were overlaid with lysosomes that were stained green and appear as yellow dots, but the KLPP^Cy5^R were almost imperceptible in lysosomes (no overlap between red and green). In contrast with strong signal of PP^Cy5^R nanoparticles in the lysosomes, after 3 h, the KLPP^Cy5^R were scattered in the cytoplasm and some reached the nucleus, indicating the non-lysosomal endocytosis pathway and confirming the membrane fusion pathway. Considering that endosomes/lysosomes have lower pH and different hydrolytic enzymes can devitalize and decompose exogenous substances including drug loaded-nanoparticles and consequently weaken or even inactivate the drug effect 40, 41, rapid and efficient endo-lysosomal escape is one of important characters of the nanoparticles. The internalization of KLPPR via a non-lysosomal pathway bypassed degradation of their payload in lysosomal traps, which was beneficial for maintaining maximal pharmacological actions.

### DN model mice

A one-time high-dose intraperitoneal injection of STZ (200 mg/kg), which is a classic method for modeling type I diabetes, was applied to rapidly damage pancreatic islet cells in this study. The DN model was established by measuring the changes in the FBG and body weight. The FBG of HC mice remained in the range of 6.3 ± 0.9 mmol/L; but increased to 18.5 ± 2.2 mmol/L at the third days after STZ injection. Diabetes was defined as FBG ≥ 15 mmol/L. FBG increased with the progression of disease and ultimately reached at the level of 23.9 ± 1.7 mmol/L. The body weight decreased markedly and was about 17.5 ± 1.2 g (24.5 ± 1.6 g in HC group), which was mainly caused by the large loss of glucose and protein through the kidneys after 5 weeks STZ injection (Figure S7), indicating that diabetes had deteriorated into DN stage 30, 31.

### *In vivo* stability and blood clearance

**Figure 5:**
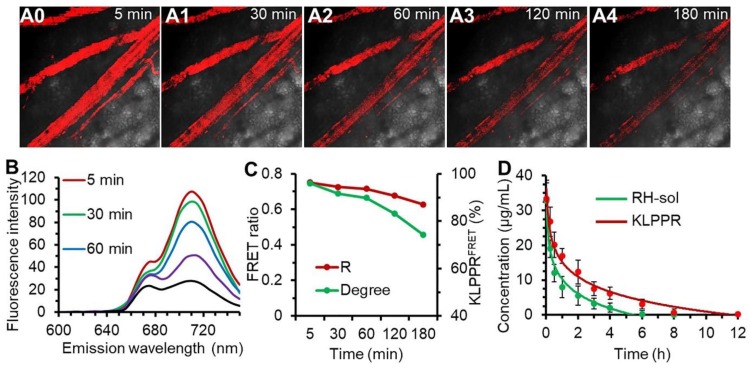
** The *in vivo* stability of KLPPR^FRET^ in bloodstream and blood clearance**. Confocal FRET fluorescence images of a mouse vein (A0-A5) and the corresponding spectra (B) at timed intervals after injection via the tail vein of KLPPR^FRET^. The FRET ratio and degree of stability was calculated from the intensities of the fluorescence at 670 nm and 710 nm (C). The blood clearance of RH-sol and KLPPR in DN mice (D).

Upon injection into the bloodstream, nanoparticles were immediately surrounded by blood components, and the *in vitro* stability of the nanoparticles were challenging by the strong frictional force between blood and endothelium wall, protein adsorption and wall-shear stress 42, 43. Shear-stress-produced drag forces and serum protein in vascular narrowing was found to induce disassembly of microscale nanoparticles and vesicles. We hypothesized that blood components and other stress might together ruin the core-shell structure and thus facilitate KLPPR dissociation. This role was also assessed using FRET. The blood microvessels in the abdomen skin were imaged for FRET fluorescence at 710 nm excited at 640 nm and the corresponding fluorescence spectra of the bloodstream were recorded using confocal microscopy (Figure 5, A0-A4 and B). At 5 min post-injection of KLPPR^FRET^, the FRET fluorescence of the blood was clearly visible, became slight weaker after 30 min and gradually dimmed out thereafter (Figure 5, A0-A4). The FRET ratio (R) was 0.75 at 5 min and maintained above 0.71 with a strong FRET peak within 60 min, and then decreased to 0.63 at 180 min. However, the degree of KLPPR was still above 75% at 180 min, indicating the good *in vivo* stability of KLPPR (Figure 5, B and C).

The RH concentration in the blood was determined using the HPLC according to the calibration curve and the pharmacokinetic parameters were measured accordingly (Figure. 4D and Table S2). As shown in Figure 4D, KLPPR was cleared much slower than RH-sol. The RH concentration of KLPPR in the blood slowly decreased to 16.92 µg/mL from the injected dose of 33.46 µg/mL after 1 h injection and then decreased to 3.19 µg/mL after 6 h, while that of RH-sol was only 24.4% of the injected dose at 1 h and rapidly decreased to 0.26 µg/mL at 6 h post-injection. The area under the curve (AUC) and elimination half-time (T_1/2_) of KLPPR were 3.17 times and 3.43 times greater than those of RH-sol, respectively (Table S2). The RH concentration of KLPPR in the kidneys was 6.27 ± 0.55 µg/mL at 12 h post-injection, while that of RH-sol was only 0.36± 0.07 µg/mL, confirming that KLPPR could improve nearly 16.42-fold increase of Rhein in kidneys (Figure S8). These results suggested that KLPPR greatly improved the blood circulation and kidney accumulation of the RH, making it possible for DN therapy by systemic administration.

### Biodistribution of KLPPR in DN model mice

**Figure 6:**
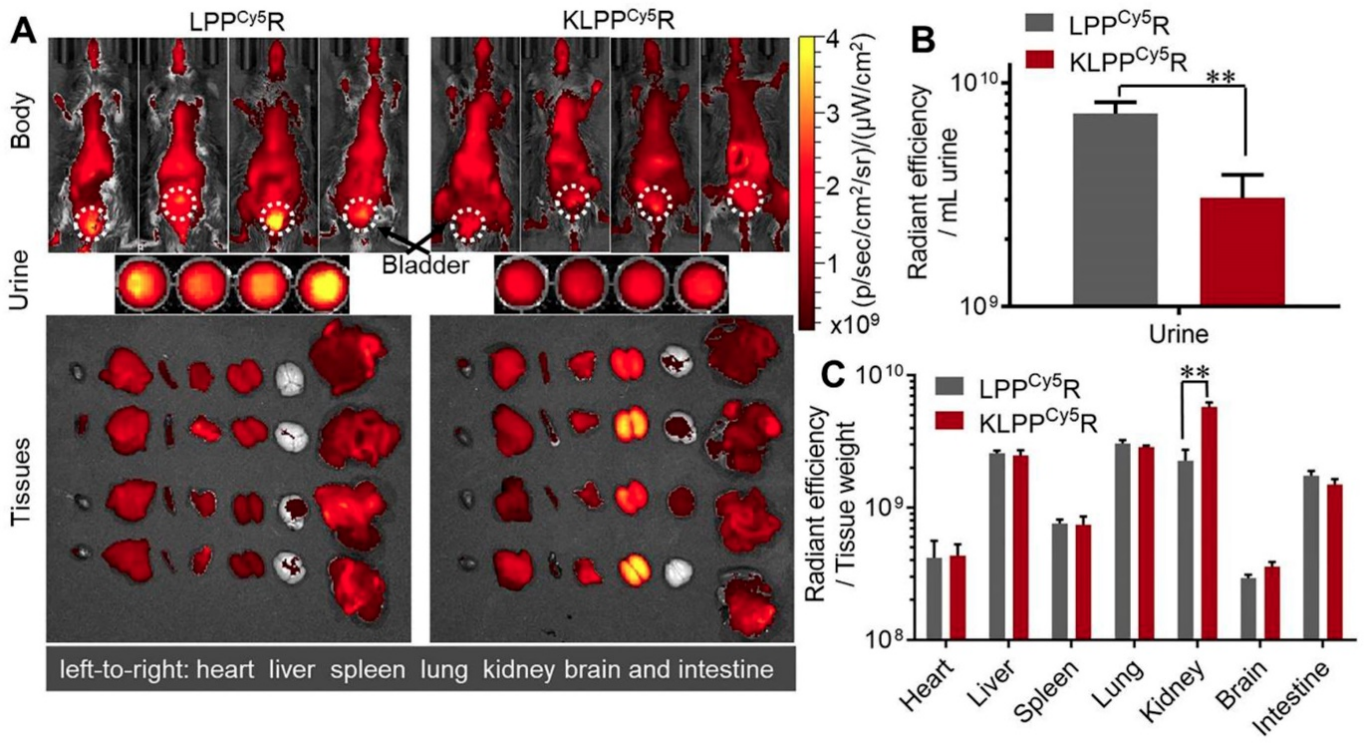
** Biodistribution of LPP^Cy5^R and KLPP^Cy5^R in DN mice via intravenous injection**. Images of whole body, urine and organs were obtained with Caliper IVIS Lumina II system (A). The quantitative analysis of the fluorescence intensity in urine (B) and organs (C) was based on images in A. The fluorescence intensity was quantized with the unit of radiant efficiency by using the Living Image^®^-4.5 software. *: *P* < 0.05, **: *P* < 0.01.

As shown in Figure 6A, LPP^Cy5^R and KLPP^Cy5^R were distributed widely throughout the body of the mice and accumulated in the major organs as a result of metabolism and clearance. Both LPP^Cy5^R and KLPP^Cy5^R were mainly distributed in the chest and abdomen of mice after 8 h. The LPP^Cy5^R showed strong fluorescence in the bladder and urine, whereas KLPP^Cy5^R exhibited enhanced fluorescence in the kidneys. The fluorescence intensity of urine and major organs were quantitatively analyzed (Figure 6B and C). The urine fluorescence intensity of LPP^Cy5^R-treated mice was 2.4-fold that of KLPP^Cy5^R mice, demonstrating the rapid excretion by urine. LPP^Cy5^R mainly distributed into liver and lung, followed by kidneys and intestine. Strikingly, fluorescence intensity in the kidneys of KLPP^Cy5^R-treated mice was 2 ~ 4 times higher than that of liver, lung and intestine, and 7 ~ 20 times than that of heart, spleen and brain. In comparison, the KLPP^Cy5^R exhibited a 2.6 times greater fluorescence intensity in kidneys than LPP^Cy5^R. These results revealed that KTP mediation could improve kidney distribution and accumulation and lower the urinary excretion by enhancement of the renal cellular uptake of KLPP^Cy5^R.

### Pharmacodynamics and therapeutics of DN model mice

**Figure 7:**
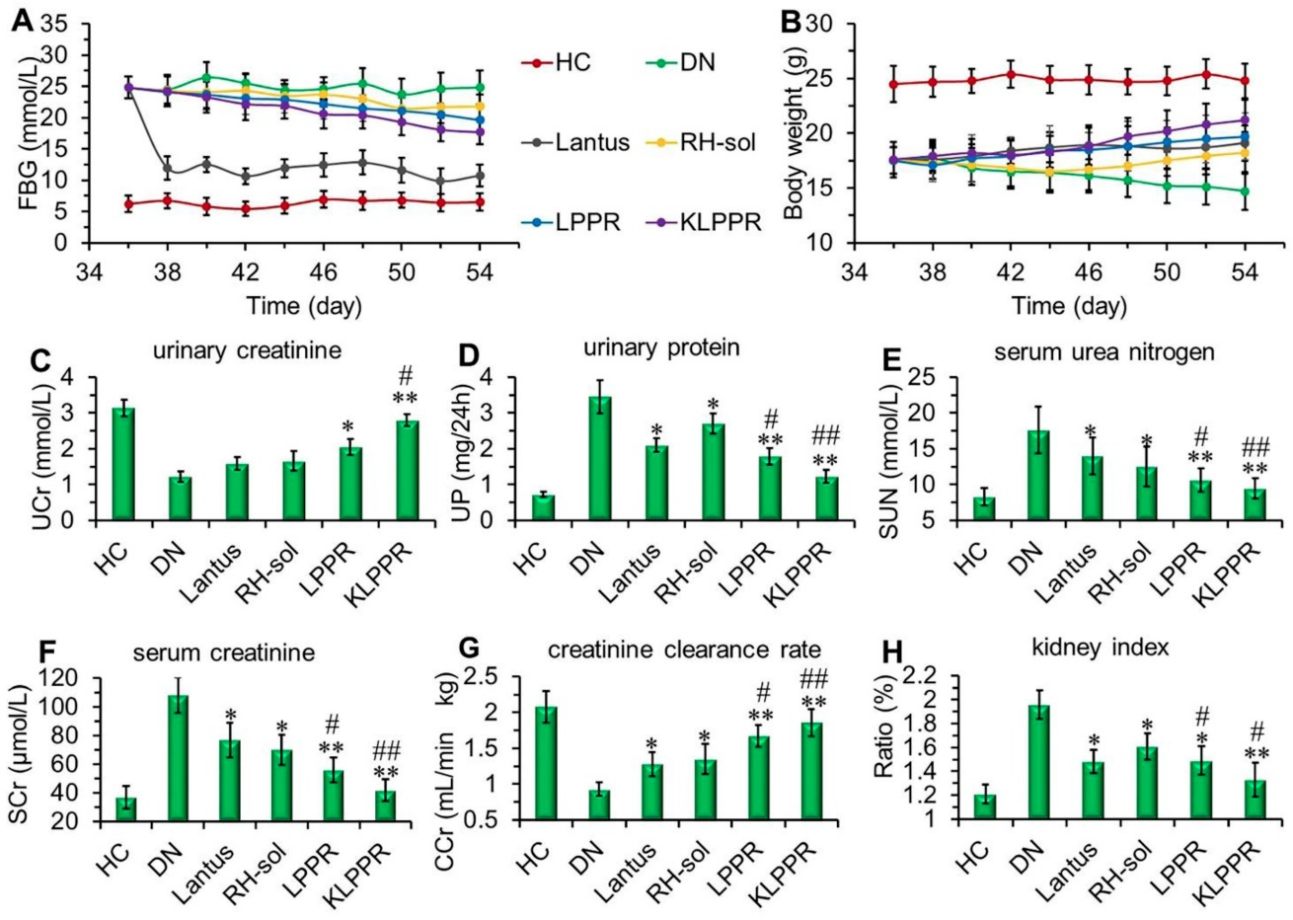
** Therapeutic efficacy and biochemical parameters in the blood and urine of DN mice after different treatments.** Changes in the FBG (A) and body weight (B) were recorded in each group during treatment. At the end of treatment, urinary creatinine (UCr, C), urinary protein (UP, D), serum urea nitrogen (SUN, E), serum creatinine (SCr, F), creatinine clearance rate (CCr, G) and kidney index (H) of the mice in each group were measured and analyzed. Comparison with DN group, * *P* < 0.05, ** P < 0.01. Comparison with RH-sol group, # *P* < 0.05, ##* P* < 0.01.

Lantus administration resulted in a sharp decrease in blood glucose and the FBG was maintained at 9~12 mmol/L (Figure 7A). FBG levels in RH-sol, LPPR and KLPPR-treated DN mice decreased to the range of 17~21 mmol/L and were slightly less than that in DN group (24 mmol/L); but these parameters were still not as good as HC group. The body weight of RH-sol treated mice began to increase after the 5^th^ injections, whereas the mice in Lantus, LPPR and KLPPR group exhibited gradual weight gaining after the first administration. At the end of the experiment, Lantus and KLPPR groups exhibited better indices of blood glucose and body weight, whereas RH-sol and LPPR groups had minor therapeutic effects (Figure 7A and 7B). The Blood and urine biochemical parameters associated with DN were measured in all groups. The UP, SUN, SCr and kidney index of the KLPPR and LPPR group were significantly lower than those of the DN group, whereas the UCr and CCr were significantly higher. The therapeutic effect was similar in Lantus group and RH-sol group as evidenced by UP, SUN, SCr, kidney index, UCr and CCr, but the effect was weaker than that in the LPPR and KLPPR (Figure 7C-H). The RH therapeutic effect was better in the KLPPR group than DN group and RH-sol group and significantly slowed down the progression from renal dysfunctions to DN in the mice. The UCr and CCr in KLPPR group were 2.79 ± 0.17 mmol/L and 1.90 ± 0.12 mL/min·kg, respectively, whereas the respective values in RH-sol group were 1.66 ± 0.27 mmol/L and 1.35 ± 0.21 mL/min·kg. The UP, SUN, SCr, and kidney index in KLPPR-treated mice were 1.23 ± 0.18 mg/24h, 9.5 ± 1.43 mmol/L, 42 ± 7.6 μmol/L, and 1.33 ± 0.14, respectively; in contrast, the respective values were 3.45 ± 0.46 mg/24h, 17.6±3.24 mmol/L, 108.7 ±12.5 μmol/L and 1.96 ± 0.13 in DN group, demonstrating the better kidney function achieved by the administration of KLPPR.

**Figure 8:**
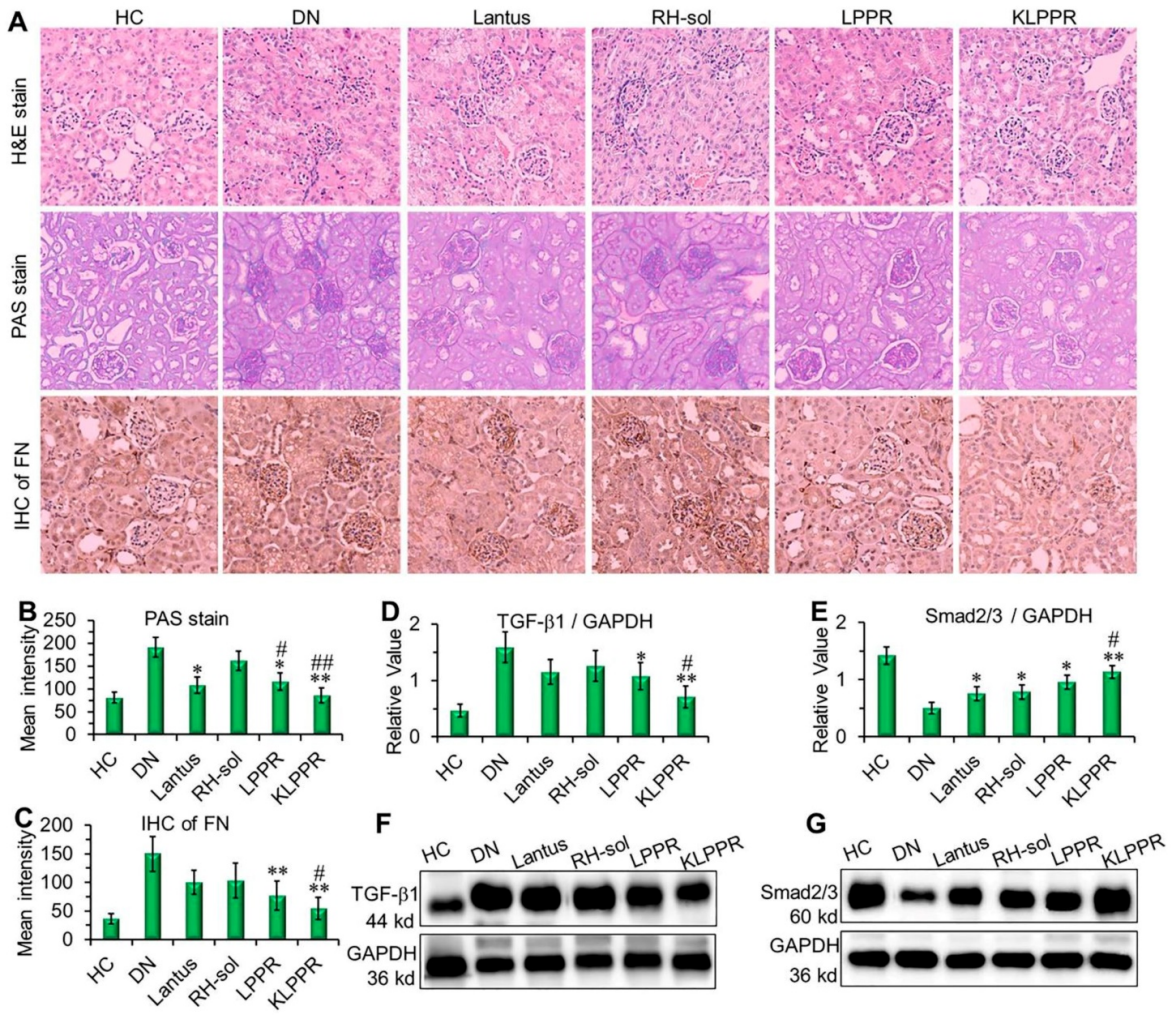
**Pathological analysis and western blotting of the kidneys.** Histological H&E staining, PAS staining, and IHC of FN were evaluated after different treatments (A). The quantitative analysis of PAS staining (B) and FN (C) were conducted by using Image J software. The expression of TGF-β1 (D, F) and Smad2/3 (E, G) were evaluated by Western blotting. Comparison with DN group, * *P* < 0.05, ** P < 0.01. Comparison with RH-sol group, # *P* < 0.05, ##* P* < 0.01.

The results of H&E staining, PAS staining and IHC of the fibronectin (FN) were evaluated to confirm DN pathological features. As shown in Figure 8A, the kidney in DN group exhibited glomerular enlargement and sclerosis, membrane matrix dilatation, expansion of the mesangial area, a thickened basement membrane, and extensive damage of the renal tubules and vascular plexus, which are typical DN complications. A greatly thickened glomerular basement membrane and accumulated mesangial matrix were observed (light purple in PAS staining) and the infiltration extended to the entire renal capsule compartment. After Lantus, RH-sol, LPPR and KLPPR treatments, the disease progression was alleviated in all drug-treated groups, unlike in the DN group. The Lantus, LPPR, and KLPPR groups exhibited considerable inhibition of glomerular enlargement, improved glomerular vascular circulation, reduced lumen volume, suppressed proliferation of the mesangial matrix and glomerular basement membrane thickening (Figure 8A and 8B). FN is a component of glomerular basement membrane and mesangial matrix and represents a major extracellular matrix protein during DN deterioration; therefore, it plays an important role in renal fibrosis and glomerular sclerosis 44, 45. The IHC images showed that glomerular membrane was stained dark brown in DN mice, suggesting a rapid increase in the secretion of FN in the glomerulus, as well as a thickened glomerular basement membrane and accumulated mesangial matrix (Figure 8A). Unlike the weak inhibition of FN expression in Lantus group, KLPPR significantly inhibited the secretion and accumulation of FN and alleviated the DN deterioration and its effect was better than that of LPPR (Figure 8A and 8C).

As DN progress, TGF-β1 and Smad2/3 are the key intermediaries in the signaling pathways of kidney damage induced by biochemical factors such as hyperglycemia and cytokines 46; TGF-β1 and Smad2/3 are closely related to abnormal renal cell proliferation, glomerular hypertrophy, extracellular matrix protein accumulation, renal interstitial fibrosis and damages to the glomerular filtration barrier 6, 46-48. Compared with DN group, a reduced expression of TGF-β1 and an up-regulated expression of Smad2/3 were observed in the LPPR and KLPPR groups, whereas Lantus only affected the Smad2/3 expression (Figure 8D-E); this indicated that the DN progression was prevented by blocking the inflammatory signaling pathways. In addition, in terms of TGF-β1 and Smad2/3 expression, KLPPR exhibited a higher pharmacological efficacy of RH than LPPR. Lantus helped to lower blood glucose levels to reduce the burden on the kidneys, whereas KLPPR regulated the abnormal expression of the proteins in the kidneys to improve renal functions. RH was loaded and delivered by KLPPR, thereby enabling kidney-targeted distribution and retention and providing highly effective DN therapy.

## Conclusion

We successfully synthesized PCL-PEI polymer and prepared RH-loaded KLPPR liponanoparticles with a yolk-shell structure consisting of PCL-PEI-based nanoparticles and a KTP modified lipid layer. KLPPR exhibited several advantages in terms of high encapsulation efficiency, drug loading content, sustained release, good stability and biocompatibility, rapid cellular uptake and endocytosis via a non-lysosomal pathway. In STZ-induced DN model, KLPPR exhibited excellent kidney-targeted distribution and retention and substantially improved the therapeutic effect of RH on DN and ameliorated the progression of DN by exploiting the two-step nanoparticular cascade of size control and enhancement of renal cellular uptake. This study provides guidance for further clinical and translational research of RH in DN therapy and offer a promising strategy for treatment of renal diseases using the nanoparticle delivery system.

## Supplementary Material

Synthetic route and ^1^H NMR spectra of the polymers; The MALDI-TOF MS spectrum and ^1^H NMR spectra of DSPE-PEG-KTP, KTP protein and DSPE-PEG-NHS; HPLC chromatograms of RH standard solution and plasma added with RH standard solution; Changes of FBG and body weight in healthy control mice and diabetic nephropathy mice within five weeks after STZ-injected; Pharmacokinetic parameters and the Rhein concentration in kidney of RH-sol and KLPPR.

## Abbreviations

APEI: acetylated polyethyleneimine
AUC_0-t_: area under curve
CMC: critical micelle concentration
CCr: creatinine clearance rate
CHEMS: cholesteryl hemisuccinate
DN: diabetic nephropathy
DSPE-PEG-KTP: KTP-modified DSPE-PEG
DOPE: 1,2-Dioleoyl-sn-glycero-3-phosphoethanolamine
DSPE-PEG: 1,2-distearyl-sn-glycerol-3-phosphoethanolamine-polyethylene glycol
EE: encapsulation efficiency
FRET: fluorescence resonance energy transfer
FN: fibronectin
FBG: fasting blood glucose
HC: healthy control group
H&E: hematoxylin and eosin staining
IHC: immunohistochemistry
KTP: kidney targeting peptide
KLPPR: kidney-targeted Rhein-loaded liponanoparticles or RH-loaded KTP-modified liposomal PCL-PEI nanoparticles
KLPP^Cy5^R: Cy5-labeled KLPPR
LPPR: RH-loaded liposomal PCL-PEI nanoparticles
LPP^Cy5^R: Cy5-labeled LPPR
LE: loading efficiency
MRT: mean retention time
PCL-PEI: polycaprolactone-polyethyleneimine
PPR: RH-loaded PCL-PEI nanoparticles
PAS: periodic acid-schiff staining
PDI: polydispersity index
PEG: polyethylene glycol
PEI: polyethyleneimine
RH: rhein
RH-sol: RH solution
RBC: red blood cells
SUN: serum urea nitrogen
SCr: serum creatinine
STZ: streptozotocin
TGF-β1: transforming growth factor-beta1
T_1/2_: elimination half-time
UCr: urinary creatinine
24h-UV: 24-hour urinary volume
24h-UPE: 24-hour urinary protein excretion
