# Supplementary material for: Kidney-targeted rhein-loaded liponanoparticles for diabetic nephropathy therapy via size control and enhancement of renal cellular uptake
Source: Theranostics. 2019 Aug 14;9(21):6191–208. doi: 10.7150/thno.37538 (PMC6735513; doi:10.7150/thno.37538)
Supplement: Supplementary file 1 — Synthetic route and 1H NMR spectra of the polymers; The MALDI-TOF MS spectrum and 1H NMR spectra of DSPE-PEG-KTP, KTP protein and DSPE-PEG-NHS; HPLC chromatograms of RH standard solution and plasma added with RH standard solution; Changes of FBG and body weight in healthy control mice and diabetic nephropathy mice within five weeks after STZ-injected; Pharmacokinetic parameters and the Rhein concentration in kidney of RH-sol and KLPPR. [file thnov09p6191s1.pdf]

# Kidney-targeted rhein-loaded liponanoparticles for diabetic nephropathy therapy via size control and enhancement of renal cellular uptake

Guowei Wang[1], Qunying Li[1], Danfei Chen[2], Bihan Wu[1], Yulian Wu[1], Weijun Tong[3], and Pintong Huang[1, *]

[1] Department of Ultrasound in Medicine, The Second Affiliated Hospital of Zhejiang University School of Medicine, Zhejiang University, Hangzhou 310009, China.

[2] Department of Pediatrics, The First Affiliated Hospital of Zhejiang Chinese Medical University, Zhejiang Chinese Medical University, Hangzhou, 310006 China.

[3] MOE Key Laboratory of Macromolecular Synthesis and Functionalization, Department of Polymer Science and Engineering, Zhejiang University, Hangzhou 310027, China.

[*] Corresponding author, huangpintong@zju.edu.cn (P. Huang)

## Supplementary Material

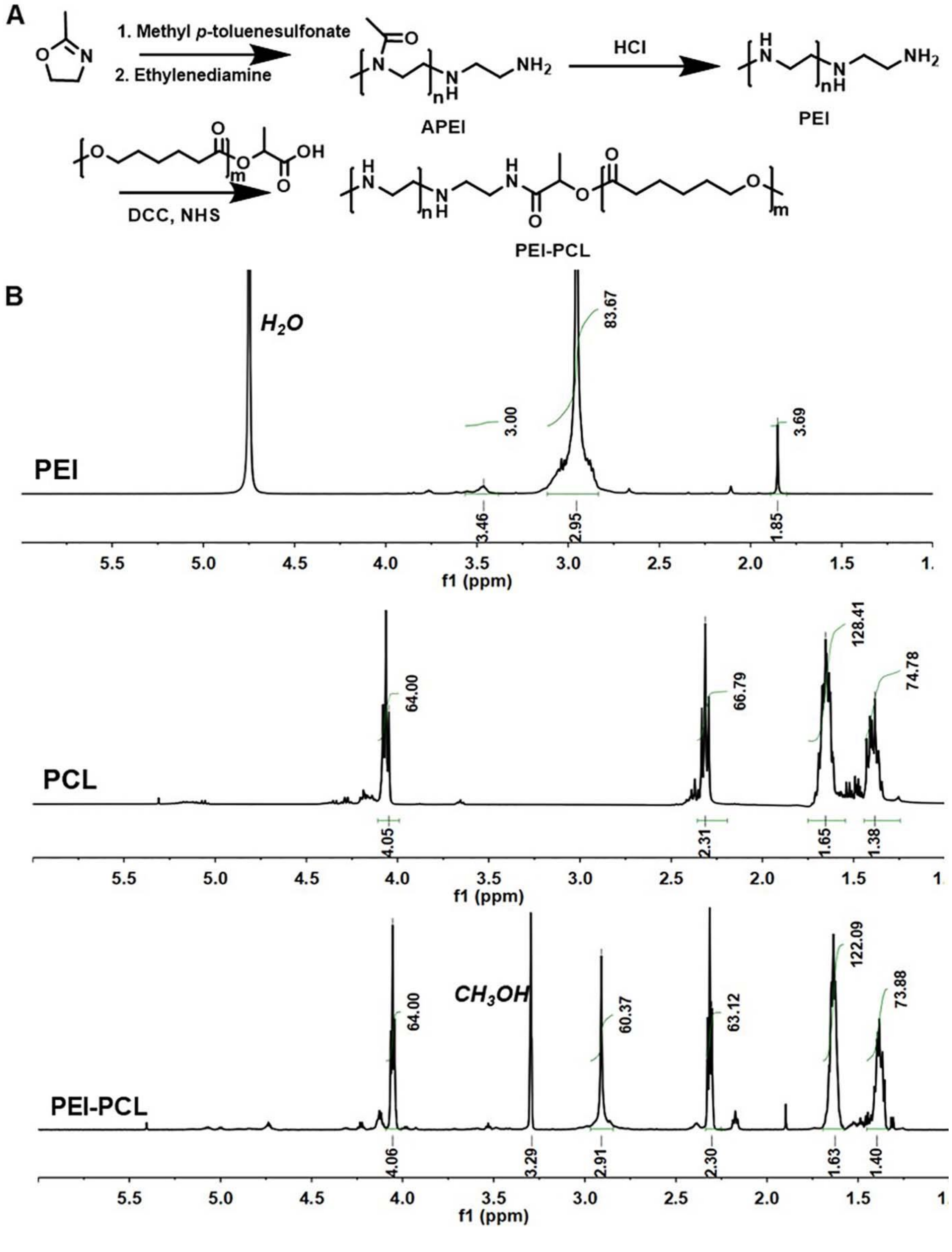


**Figure S1.** Synthetic route (A) and $^1$H NMR spectra of the polymers (B).

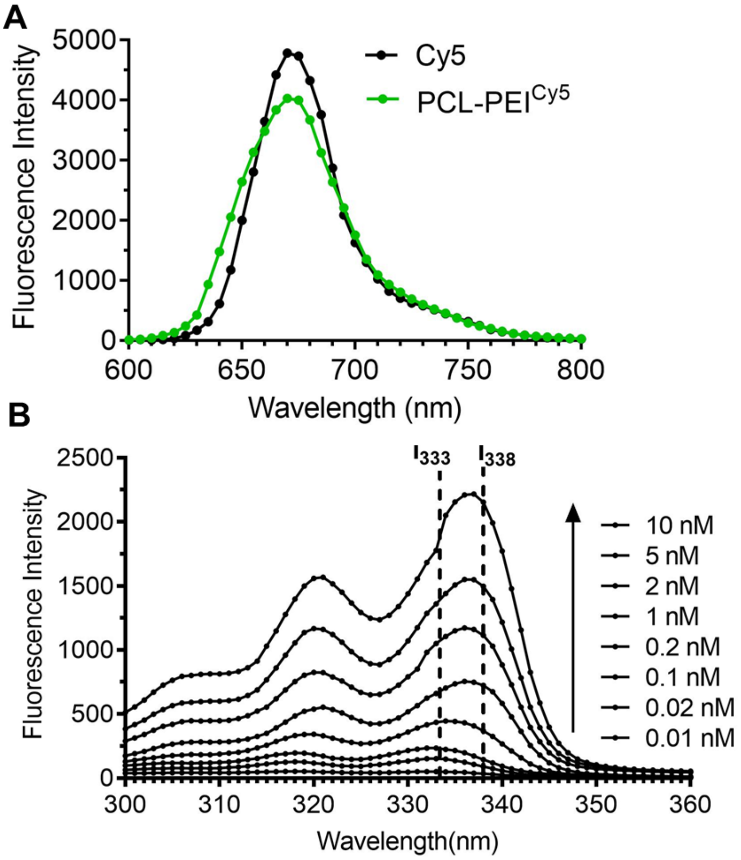


**Figure S2.** Fluorescent scanning spectra of Cy5 labeled PCL-PEI (A). Fluorescence intensity of pyrene against various concentrations of polymers (B).

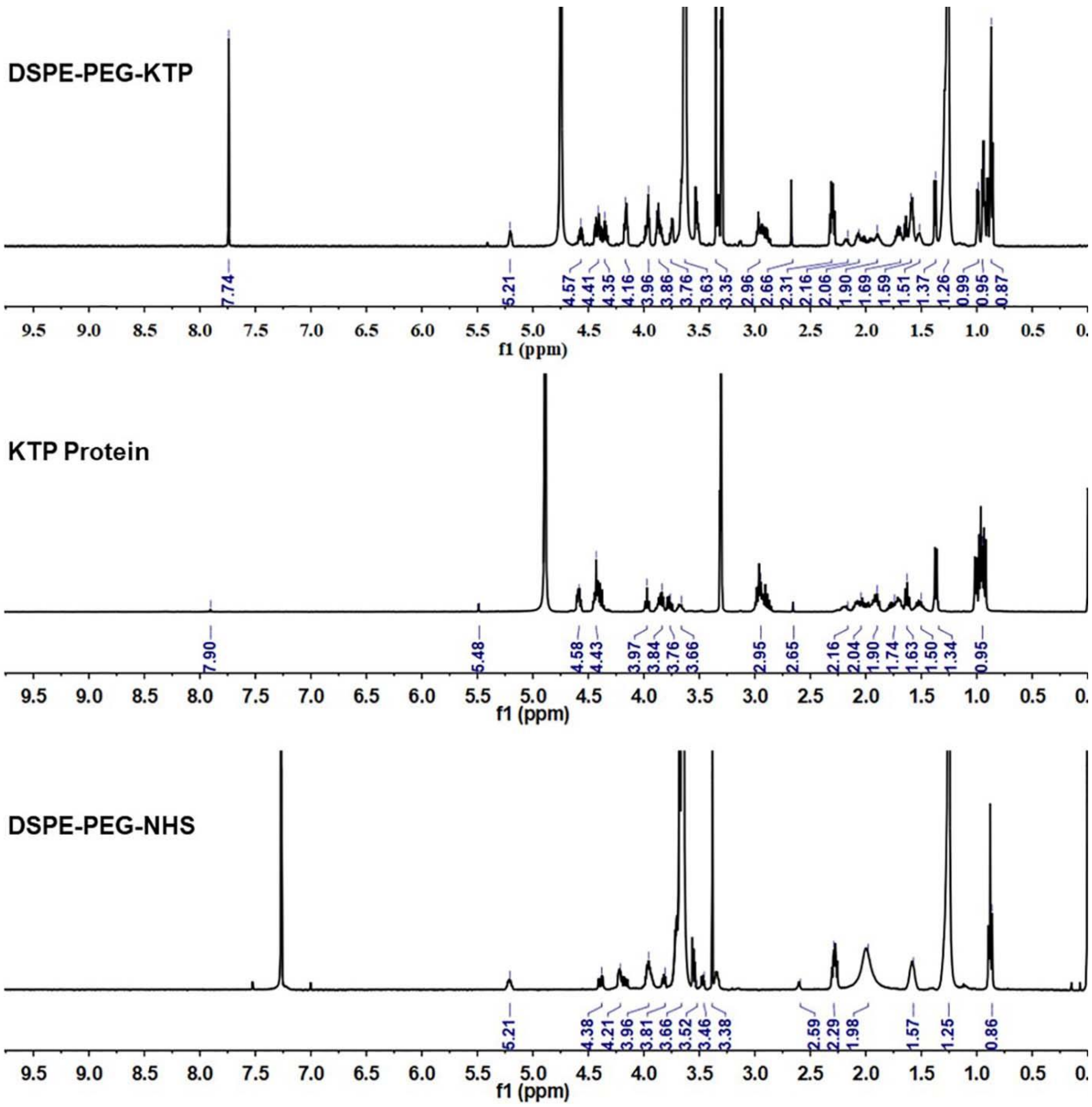


**Figure S3.** $^{1}H$ NMR spectra of DSPE-PEG-KTP, KTP protein and DSPE-PEG-NHS.

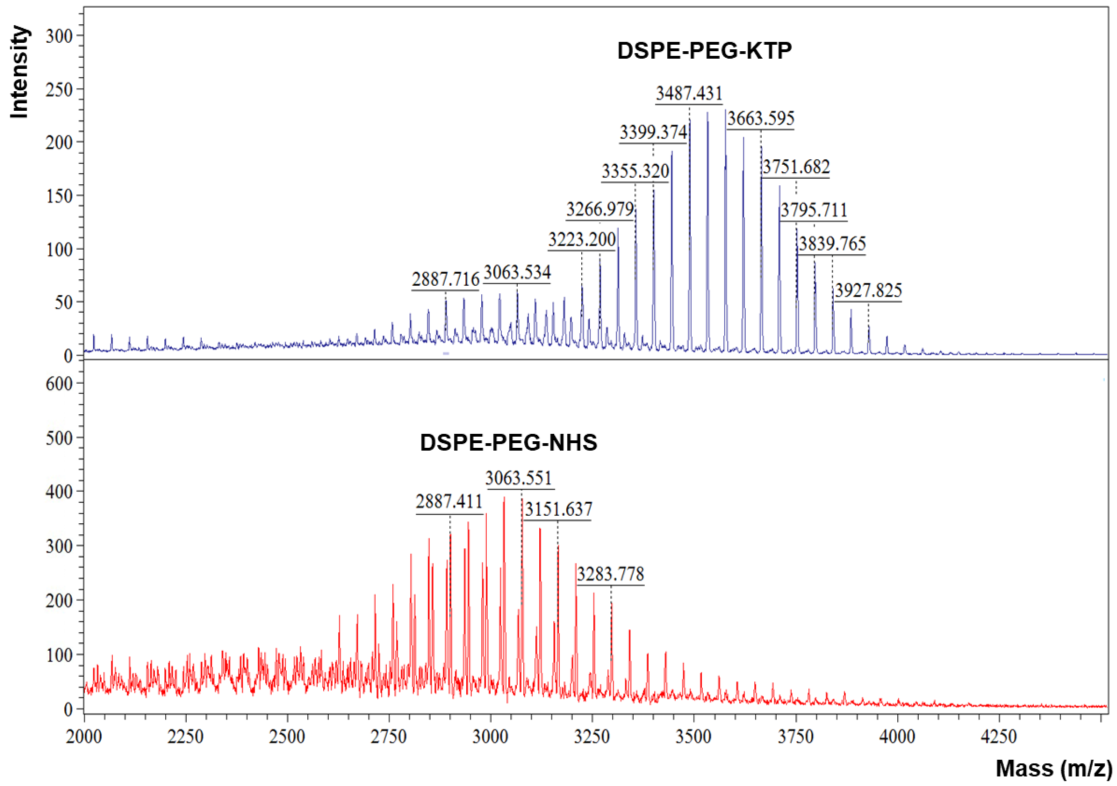


**Figure S4.** The MALDI-TOF-MS spectrum of DSPE-PEG-KTP and DSPE-PEG-NHS.

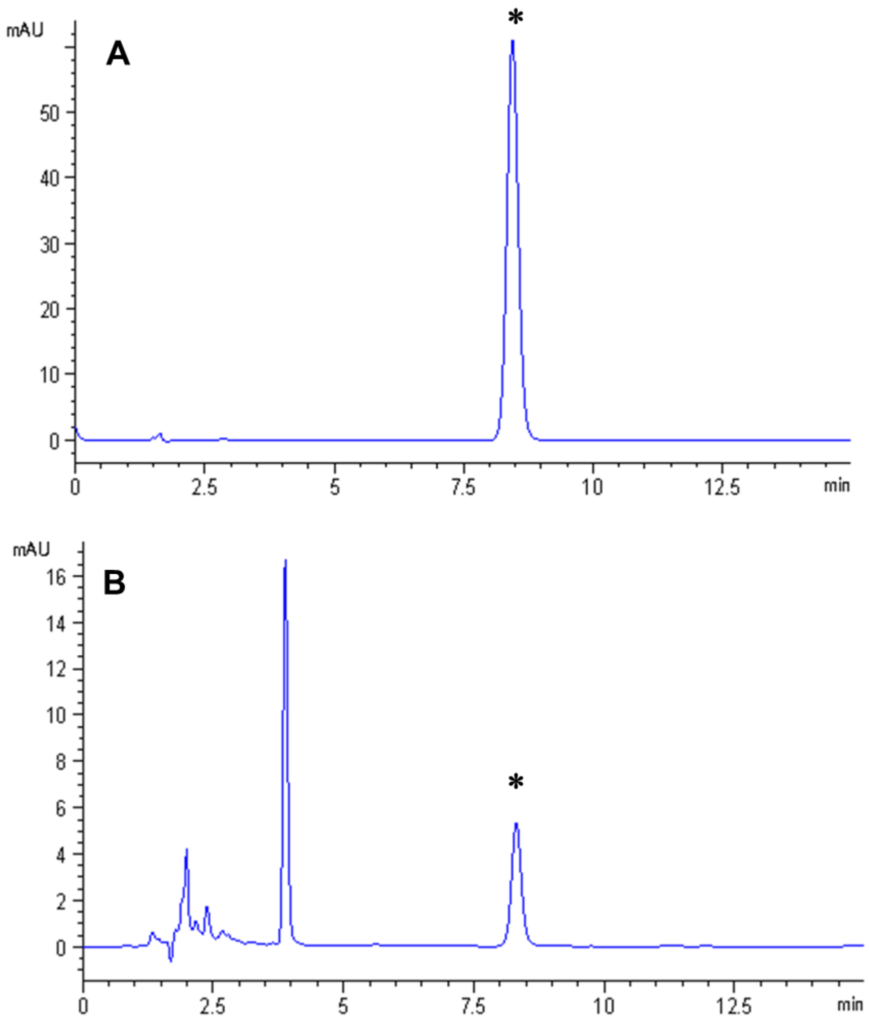


**Figure S5.** HPLC chromatograms of RH standard solution (A) and plasma added with RH standard solution (B). The retention time of RH was 8.44 min. ∗ indicated the chromatograph peak of RH.

**Table S1.** Release kinetics of KLPPR in PBS (pH 7.4).

| Model | Regression equation | $R^2$ |
|---|---|---|
| Zero-order | Q = 0.0117t + 0.1867 | 0.6105 |
| First-order | ln(1-Q) = -0.0191t - 0.2168 | 0.7218 |
| Higuchi | Q = 0.979$t^{1/2}$ + 0.0648 | 0.8655 |

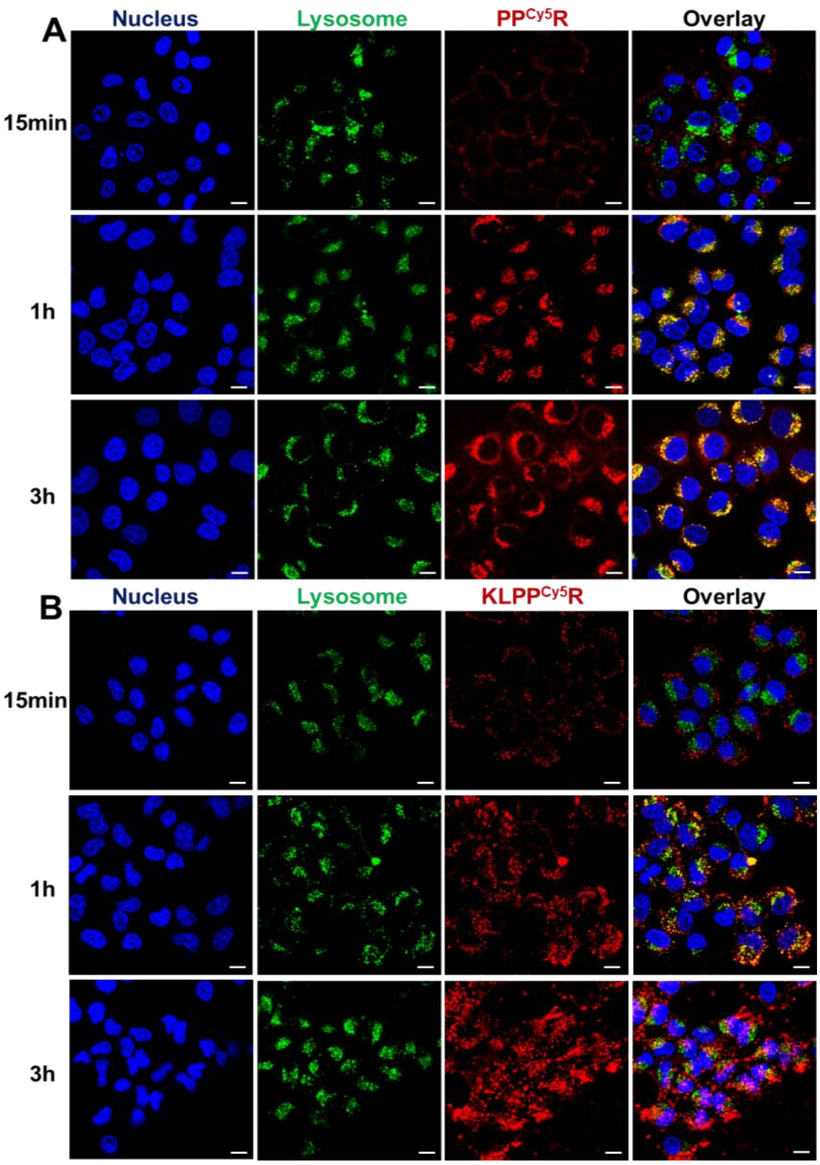


**Figure S6.** Subcellular distribution of (A) PP$^{Cy5}$R and (B) KLPP$^{Cy5}$R. HK-2 cells were imaged with a CLSM and the nuclei were stained with Hoechst 33342 (blue), the lysosomes were labeled with LysoTracker® Green DND26 (green) and the nanoparticles were labeled with Cy5 (red). The scale bar is 50 μm.

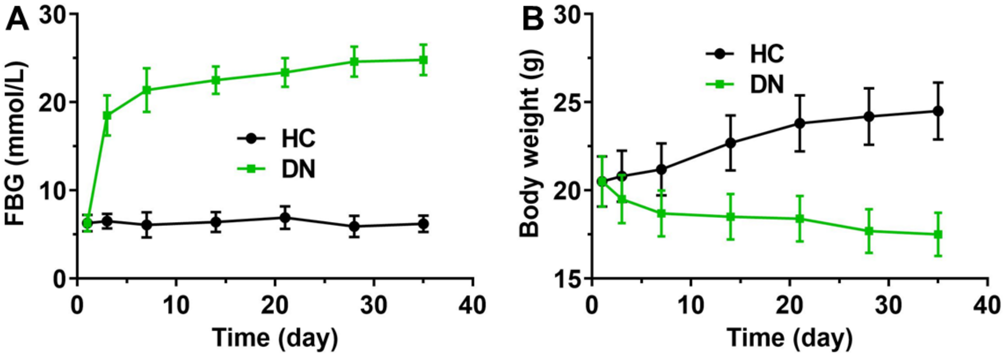


**Figure S7.** FBG (A) and body weight (B) changes of healthy control mice and diabetic nephropathy mice in five weeks after STZ-injected.

**Table S2**. Pharmacokinetic parameters of RH-sol and KLPPR (intravenously, dose equivalent to RH 5 mg/kg, 4 mice in each group). Data were presented as mean ± SD.

| Parameters | Unit | RH-sol | KLPPR |
|---|---|---|---|
| $T_{1/2}$ | h | 0.42 ± 0.17 | 1.44 ± 0.28 |
| CL | (μg)/(μg/mL)/h | 5.09 ± 1.48 | 1.60 ± 0.33 |
| $AUC_{0-t}$ | μg /mL·h | 19.64 ± 3.55 | 62.26 ± 7.29 |
| MRT | h | 0.65 ± 0.13 | 2.18 ± 0.45 |

$T_{1/2}$, CL, $AUC_{0-t}$ and MRT refer to elimination half-time, clearance, area under curve and mean retention time respectively.

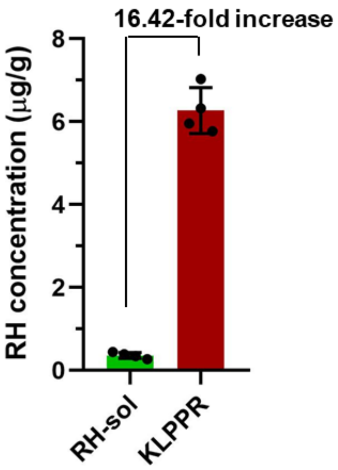


**Figure S8.** The concentration of Rhein in kidney at 12 h post-injection in diabetic

nephropathy mice. The DN mice were intravenously injected with RH-sol and KLPPR (dose equivalent to RH 5 mg/kg, 4 mice in each group).
